# Pathological ASXL1 Mutations and Protein Variants Impair Neural Crest Development

**DOI:** 10.1016/j.stemcr.2019.03.006

**Published:** 2019-04-18

**Authors:** Friederike Matheus, Ejona Rusha, Rizwan Rehimi, Lena Molitor, Anna Pertek, Miha Modic, Regina Feederle, Andrew Flatley, Elisabeth Kremmer, Arie Geerlof, Valentyna Rishko, Alvaro Rada-Iglesias, Micha Drukker

**Affiliations:** 1 Institute for Stem Cell Research, Helmholtz Zentrum München GmbH, 85764 Neuherberg, Germany; 2 Institute for Stem Cell Research, iPSC Core Facility, Helmholtz Zentrum München GmbH, 85764 Neuherberg, Germany; 3 Center for Molecular Medicine Cologne (CMMC), 50931 Köln, Germany; 4 The Francis Crick Institute, London NW1 1AT, UK; 5 Department for Neuromuscular Diseases, UCL Queen Square Institute of Neurology, London WC1N 3BG, UK; 6 Institute for Diabetes and Obesity, Monoclonal Antibody Core Facility, Helmholtz Zentrum München GmbH, 85764 Neuherberg, Germany; 7 Institute of Molecular Immunology, Helmholtz Zentrum München GmbH, 85764 Neuherberg, Germany; 8 Institute of Structural Biology, Protein Expression and Purification Facility, Helmholtz Zentrum München GmbH, 85764 Neuherberg, Germany

**Keywords:** ASXL1, neural crest, Bohring-Opitz syndrome, Polycomb, ZIC1

## Abstract

The neural crest (NC) gives rise to a multitude of fetal tissues, and its misregulation is implicated in congenital malformations. Here, we investigated molecular mechanisms pertaining to NC-related symptoms in Bohring-Opitz syndrome (BOS), a developmental disorder linked to mutations in the Polycomb group factor *Additional sex combs-like 1* (*ASXL1*). Genetically edited human pluripotent stem cell lines that were differentiated to NC progenitors and then xenotransplanted into chicken embryos demonstrated an impairment of NC delamination and emigration. Molecular analysis showed that *ASXL1* mutations correlated with reduced activation of the transcription factor *ZIC1* and the NC gene regulatory network. These findings were supported by differentiation experiments using BOS patient-derived induced pluripotent stem cell lines. Expression of truncated ASXL1 isoforms (amino acids 1–900) recapitulated the NC phenotypes *in vitro* and *in ovo*, raising the possibility that truncated ASXL1 variants contribute to BOS pathology. Collectively, we expand the understanding of truncated ASXL1 in BOS and in the human NC.

## Highlights

• Generation of human pluripotent stem cell models for Bohring-Opitz syndrome
• *ASXL1* mutations perturb neural crest (NC) emigration *in vitro* and *in vivo*
• *ASXL1* mutations correlate with impaired activation of *ZIC1*
• Expression of truncated ASXL1 variants mirrors NC perturbation *in vitro* and *in vivo*

## Teaser

In this study, Drukker and colleagues developed human pluripotent stem cell models for the rare congenital disorder Bohring-Opitz syndrome, which is caused by mutations in the Polycomb factor *ASXL1*. In these lines, they found impaired neural crest emigration *in vitro* and *in vivo* and link this phenotype to impaired activation of *ZIC1*.

## Introduction

The neural crest (NC) is an embryonic progenitor population that gives rise to multiple derivatives, including craniofacial cartilage and bones and peripheral neurons, many of which are associated with birth defects such as craniofacial and cardiac malformations (Etchevers et al., 2006, Mayor and Theveneau, 2013, Snider and Mishina, 2014). Animal studies have shown that initiation of NC development at the neural plate border, as well as subsequent delamination, migration, and terminal differentiation of the NC progenitors, are orchestrated by specific gene regulatory networks (GRNs) (Simoes-Costa and Bronner, 2015). Advancing the understanding of these GRNs in human NC development necessitates modeling by pluripotent stem cells, in particular for investigating the epigenetic mechanisms that regulate them (Bajpai et al., 2010). Nevertheless, the involvement of mutations in histone-modifying enzymes in pathological NC development has not yet been studied.

Bohring-Opitz syndrome (BOS) is a severe congenital disorder associated with *de novo* mutations in the *Additional sex combs-like 1* (*ASXL1*) gene, characterized by symptoms that include developmental delay and musculoskeletal and neurological features (Hastings et al., 2011). ASXL1 is a co-factor of the Polycomb repressive complex 2 (PRC2), and loss of function (Abdel-Wahab et al., 2012) and expression of dominantly acting truncated ASXL1 variants (Balasubramani et al., 2015, Guo et al., 2018) have been associated with transcriptional misregulations in hematological disorders. Whether and how these mechanisms relate to the molecular mechanism of BOS is unknown.

Here, we reasoned that craniofacial malformations such as palate anomalies indicate a potential perturbation of NC development in BOS (Hastings et al., 2011). By analyzing a comprehensive set of genetically edited human embryonic stem cell (hESC) and patient induced pluripotent stem cell (iPSC) lines that we differentiated into NC progenitors, we identified transcriptional perturbations associated with defects in NC development, which we propose provide a putative mechanism for the craniofacial and plausibly other symptoms in BOS patients.

## Results and Discussion

### Generation of Human Pluripotent Stem Cell Models for BOS

**Figure 1:**
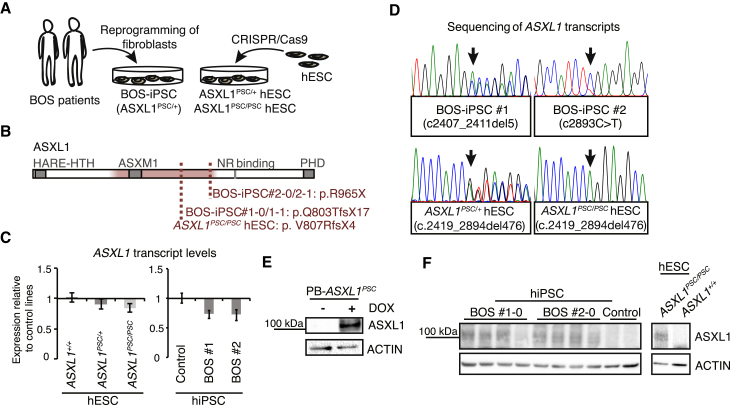
Human Pluripotent Stem Cell Models for Bohring-Opitz Syndrome (A) Scheme illustrating the generation of human pluripotent stem cell lines carrying premature stop codon (PSC) mutations in *ASXL1*. (B) Scheme of human *ASXL1* protein showing annotated domains (NR, nuclear receptor), and locations of mutations frequently reported in BOS patients (red tinted sector) and present in BOS-iPSC and *ASXL1*^*PSC/PSC*^*/ASXL1*^*PSC/+*^ hESC clones. (C) Expression of *ASXL1* in BOS-iPSC lines and *ASXL1*^*PSC/PSC*^*/ASXL1*^*PSC/+*^ hESC clones relative to the respective iPSC/hESC control lines using primers targeting exon 4 (mean ± SEM, n ≥ 3 different clones/passages). (D) Sequences of reverse transcribed *ASXL1* transcripts from *ASXL1* mutant lines. (E and F) Representative blotting (n = 3–5 independent experiments) of ASXL1, using a monoclonal antibody raised against the N terminus, in an hESC line overexpressing a truncated ASXL1 variant (PB*-ASXL1*^*PSC*^) (E) and in human iPSC and hESC lines (F). See also Figure S1.

We obtained dermal fibroblasts from two BOS patients who carry monoallelic premature stop codons (PSCs) in the terminal exon of *ASXL1* (Magini et al., 2012). Patient fibroblasts were reprogrammed using two techniques, by episomal plasmids and modified mRNAs, resulting in the derivation of four BOS-iPSC lines (Figure 1A, Table S1, Figure S1A). In addition, we used CRISPR/Cas9 to excise a 500 bp region from the terminal exon of *ASXL1* in hESCs, generating clones that harbor heterozygous and homozygous BOS patient-like PSC mutations (*ASXL1*^*PSC/+*^ and *ASXL1*^*PSC/PSC*^; Figure 1A, Table S1, Figures S1B and S1C). BOS-iPSC and edited hESC lines expressed canonical pluripotency markers and displayed colony morphology comparable with control lines, which were the parental *ASXL1*^*+/+*^ hESC line and two control iPSC lines, respectively (Figure S1A and S1C–S1G, Table S1).

Plausible molecular mechanisms of BOS include *ASXL1* haploinsufficiency and/or expression of truncated ASXL1 (Figure 1B). Testing the level of *ASXL1* transcripts in undifferentiated cells did not reveal a difference between BOS-iPSC, *ASXL1*^*PSC/+*^, and *ASXL1*^*PSC/PSC*^ clones and the respective control lines (Figure 1C). We confirmed the presence of mutant *ASXL1* transcripts in the genetically edited hESC clones, and in both BOS-iPSC lines #1-0 and #2-0 by Sanger sequencing (Figure 1D). Furthermore, we detected comparable levels of the normal and mutant transcripts using a primer set specifically targeting the 5-bp deletion in BOS-iPSC lines #1-0/#1-1 (Figure S1H). To further investigate the expression of truncated ASXL1 variants, we raised a monoclonal antibody targeting the N terminus of ASXL1, which by western blotting detected a truncated ASXL1 variant that was overexpressed in undifferentiated hESCs (PB-*ASXL1*^*PSC*^; Table S1, Figure 1E). This antibody also detected putative truncated protein isoforms in BOS-iPSC lines #1 and #2, and *ASXL1*^*PSC/PSC*^ hESC clones (Figures 1F and S1I), albeit at varying levels (Figure S1J). Collectively, this indicates that expression of truncated ASXL1 variants is a possible mode of misregulation in BOS.

### *ASXL1* Mutations Perturb NC Development

**Figure 2:**
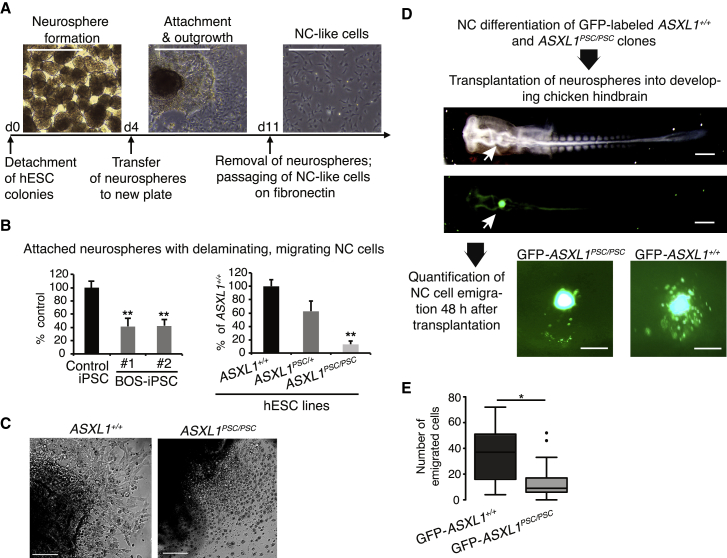
*ASXL1* Mutations Impair Differentiation of Neural Crest Progenitors (A) Timeline of neural crest (NC) differentiation protocol and bright-field images during *in vitro* differentiation of *ASXL1*^*+/+*^ hESCs. Scale bar, 500 μm. (B) Percentage of attached neurospheres with emigrating cells at day 7 of NC differentiation of BOS-iPSC lines and *ASXL1*^*PSC/PSC*^/*ASXL1*^*PSC/+*^ clones (mean ± SEM, n = 3–13 different passages/clones and independent experiments, all relative to respective control lines; ^∗∗^p<0.01). (C) Representative bright-field images of neurospheres at day 7 of NC differentiation. Scale bar, 100 μm. (D) Workflow of neurosphere transplantation experiments and representative bright-field and GFP images of chicken embryos directly (upper panel) and 48 h (lower panel) after transplantation of neurospheres (white arrows: exemplary transplanted GFP-*ASXL1*^*+/+*^ neurosphere; dorsal view). Scale bar, 500 μm (chicken embryo) and 200 μm (neurospheres). (E) Quantification of migrating cells from experiments in (D); *ASXL1*^*+/+*^, n = 9; *ASXL1*^*PSC/PSC*^, n = 21 embryos (mean ± SEM, ^∗^p = 0.037). Single dots indicate outliers. See also Figure S2.

Craniofacial symptoms that are common in BOS patients prompted us to investigate a possible connection to the NC lineage in the hESC and iPSC models. We utilized a differentiation protocol that generated delaminating NC-like cells (Bajpai et al., 2010) from neuroepithelial spheres (neurospheres; Figure 2A), which expressed the characteristic NC markers p75NTR, *SOX9*, *SOX10*, *TFAP2A*, *PAX3*, and *SNAI2* (Lee et al., 2007, Simoes-Costa and Bronner, 2015) and the proliferation marker KI67 (Figures S2A and S2B). Moreover, in accordance with the expected potency of NC progenitors, they readily gave rise to putative mesenchymal stem cells (MSCs) (Noden and Trainor, 2005) that exhibited spindle-shaped morphology, expression of consensus MSC surface markers CD73, CD90, and CD105 (Ramos et al., 2016), and terminally differentiated into osteoblasts and adipocytes (Figures S2C–S2E).

We noted upregulation of *ASXL1* and *ASXL3,* but not of *ASXL2*, in NC progenitors (Figure S2F), indicating specific roles of the two genes during NC differentiation, which was further supported by co-expression of ASXL1 and the NC regulator TFAP2A (Figure S2G). Importantly, neurospheres derived from BOS-iPSC lines with heterozygous *ASXL1* mutations displayed reduced attachment and diminished emigration of NC cells in comparison with control iPSC-derived neurospheres (Figure 2B, left panel). In the genetically edited hESC lines, we observed a similar reduction in neurosphere attachment and NC emigration, an effect that was statistically significant only in homozygous *ASXL1*^*PSC/PSC*^ clones (Figure 2B, right panel), with the presence of dead, floating cells surrounding the neurospheres (Figures 2C and S2H). *ASXL1* mutations did not negatively affect the propensity to form neurospheres; conversely, an increase in the total number of neurospheres was observed in lines harboring mutant *ASXL1* (Figure S2I). Thus, *ASXL1* mutations perturbed the generation and/or delamination of the NC *in vitro,* potentially via defective induction or specification already at the neurosphere stage.

To further test the emigration of NC cells *in vivo*, we performed orthotopic xenotransplantation of neurospheres obtained from eGFP-labeled *ASXL1*^*PSC/PSC*^ and *ASXL*^*+/+*^ hESC lines (Table S1) into Hamburger-Hamilton (HH) stage 10 chicken embryos (Figure 2D). Neurospheres derived from *ASXL1*^*PSC/PSC*^ hESC clones exhibited a significant reduction in the number of emigrating cells compared with neurospheres derived from the parental *ASXL1*^*+/+*^ hESC line (Figures 2D and 2E), an effect that was not due to size differences between control and mutant neurospheres (Figure S2J). Cells that emigrated from the neurospheres of both lines transversed a similar distance (Figure S2K). Collectively, these results indicate a connection between *ASXL1*, NC development, and BOS.

### ASXL1 Mutations Impair Activation of *ZIC1* and the NC Gene Regulatory Network

To delineate mechanisms underlying the paucity and migration defect of the NC progenitors, we conducted global RNA sequencing. This revealed mutually exclusive clustering of samples derived from NC progenitors harboring the *ASXL1*^*PSC/PSC*^ genotype and the parental control cells (Figure S3A).

**Figure 3:**
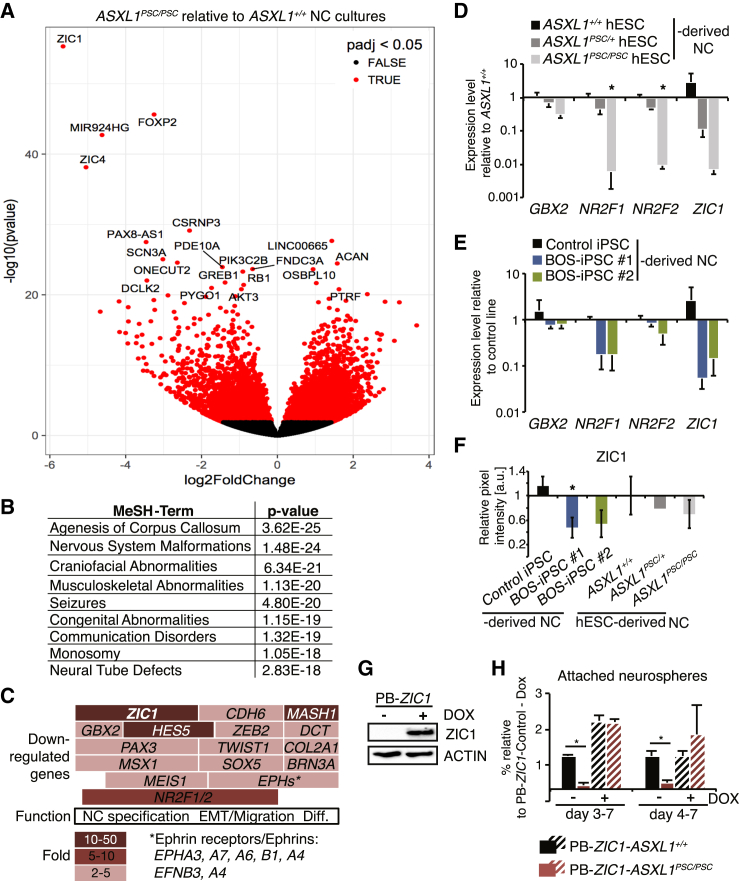
Misregulation of Gene Networks Associated with NC Development and BOS Symptoms in *ASXL1* Mutant Lines (A) Volcano plot exhibiting differentially expressed genes in *ASXL1*^*PSC/PSC*^ compared with *ASXL1*^*+/+*^ day 7 NC progenitors (total mRNA sequencing; n = 3 *ASXL1*^*+/+*^ and n = 7 *ASXL1*^*PSC/PSC*^ samples, different clones and independent differentiation experiments). (B) Most significant Medical Subject Headings (MeSH) terms associated with downregulated gene sets (p_adj_ < 0.05) in (A). (C) Diagram of genes with annotated functions in NC development and their degree of misregulation (p_adj_ < 0.05), based on (A). Diff., terminal differentiation. (D and E) Relative expression levels of canonical NC genes in day 7 NC differentiation cultures of *ASXL1*^*PSC*^ hESC lines (D) and BOS-iPSC lines (E) compared with respective control lines (mean ± SEM, n = 3–6 different clones/passages; *NR2F1* in iPSC control, n = 2 different passages; ^∗^p < 0.05). (F) Quantification of ZIC1 protein levels in NC cultures (day 7) derived from cell lines as in (D and E) compared with the respective control lines (based on western blotting, n = 3–4 different clones/passages, mean ± SEM, ^∗^p < 0.05). (G and H) Rescue of the NC differentiation defect by ectopic expression of *ZIC1*. (G) Detection of ZIC1 overexpression in DOX-treated PB-*ZIC1*-*ASXL1*^*+/+*^ by western blotting. (H) Analysis of attached neurospheres with emigrating cells in PB-*ZIC1*-*ASXL1*^*+/+*^ and PB-*ZIC1*-*ASXL1*^*PSC/PSC*^ lines at day 7, −/+ DOX treatment to overexpress *ZIC1* starting at day 3 (n = 2 and 3 independent experiments) or day 4 (n = 3 independent experiments). All data shown as mean ± SEM, ^∗^p < 0.05. See also Figure S3.

Strikingly, the neural plate border transcription factors *ZIC1* and *ZIC4* (Merzdorf, 2007), which are an immediately adjacent gene pair, were among the most negatively regulated transcripts in the *ASXL1*^*PSC/PSC*^ NC progenitors (Figure 3A). This substantiated the link to BOS and related syndromes, because heterozygous deletion of the *ZIC1*/*ZIC4* locus is associated with Dandy-Walker malformation (Grinberg et al., 2004), a defect in cerebellar development that is observed in BOS patients (Hastings et al., 2011). Moreover, negatively regulated genes in the dataset were significantly associated with several disorders and malformations found in BOS, including agenesis of the corpus callosum, craniofacial dysmorphisms, musculoskeletal abnormalities, and seizures (Figure 3B).

*ZIC1*, which was 50-fold reduced in *ASXL1*^*PSC/PSC*^ NC cultures, is at the apex of the GRN controlling NC induction (Simoes-Costa and Bronner, 2015). Accordingly, transcription factors that promote further specification, delamination, and migration of the NC were also negatively regulated in the *ASXL1*^*PSC/PSC*^ NC cells, including *PAX3*, *GBX2*, *MSX1, NR2F1/2, ZEB2*, *COL2A1*, *TWIST1*, *SOX5*, Cadherins, and Ephrins and their cognate receptors (Rada-Iglesias et al., 2012, Simoes-Costa and Bronner, 2015) (Figures 3C, S3B, and S3C, Table S2). qPCR, performed to confirm the transcriptional changes for a subset of NC genes, demonstrated significant downregulation in the *ASXL1*^*PSC/PSC*^ clones and mild reduction in the patient lines (Figures 3D and 3E), and ZIC1 protein levels were significantly lower in NC cultures derived from one BOS-iPSC line (Figures 3F and S3D). To further analyze the connection of the *ASXL1*^*PSC/+*^ ESC line to NC defects, we performed 3′ mRNA sequencing of NC cultures derived from the heterozygous *ASXL*^*PSC/+*^ clones and control hESCs. This revealed mutually exclusive clustering of the two genotypes and a significant 28-fold downregulation of *ZIC1* in *ASXL*^*PSC/+*^ NC progenitors (p_adj_ < 0.05; Figures S3E–S3G). Collectively, this supports a link between BOS-associated mutations in *ASXL1* and *ZIC1* misregulation.

To investigate whether reduced *ZIC1* levels could indeed underlie the observed NC differentiation phenotypes, we stably integrated an inducible *ZIC1* transgene in *ASXL1*^*+/+*^ and *ASXL1*^*PSC/PSC*^ hESC clones (Figure 3G, Table S1). Strikingly, overexpression of *ZIC1* during NC differentiation restored the attachment and delamination phenotype of NC-like cells from *ASXL1*^*PSC/PSC*^ neurospheres (Figure 3H). These results collectively indicate that impairment of ZIC1 activation contributes to the observed differentiation defects when *ASXL1* is mutated.

### Expression of Truncated ASXL1 Recapitulates NC Developmental Effects

**Figure 4:**
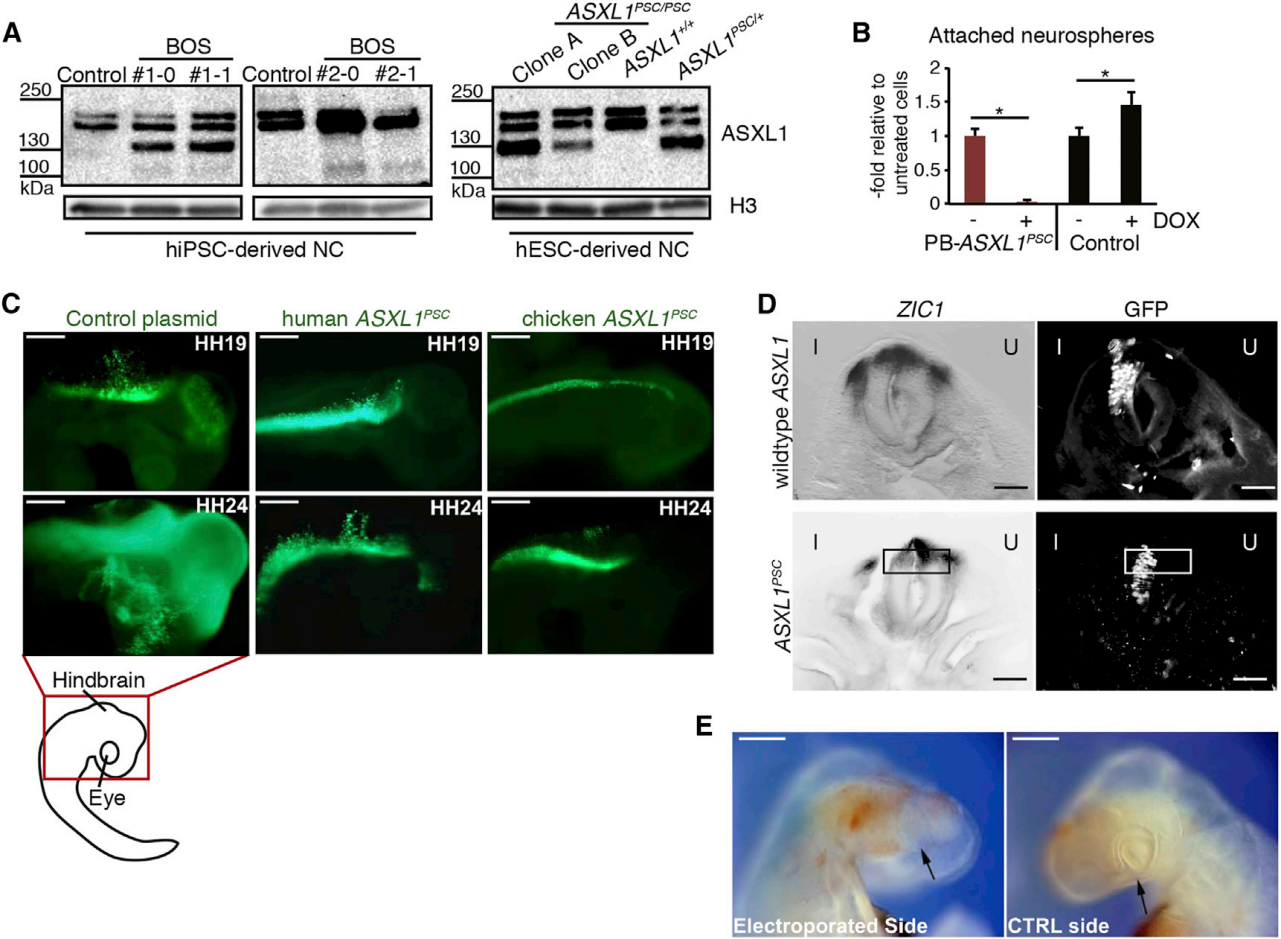
Expression of Truncated ASXL1 Impairs NC Cell Emigration and *ZIC1* Expression (A) Detection of ASXL1 by western blotting in samples of day 7 NC cultures (monoclonal antibody as in Figures 1E and 1F). (B) Percentage of attached neurospheres with emigrating cells derived at day 7 from DOX-treated *ASXL1*^*+/+*^ hESCs and PB-*ASXL1*^*PSC*^ hESCs, overexpressing truncated ASXL1, relative to untreated samples (mean ± SEM, n = 3 independent experiments, ^∗^p < 0.05). (C) Representative fluorescent images of chicken embryos 48 h after electroporation with plasmids expressing GFP or GFP coupled to truncated chicken or human *ASXL1*^*PSC*^ (n = 10 embryos each); the red rectangle indicates displayed head/hindbrain region. Scale bars, 200 μm. (D) Expression pattern of *ZIC1* analyzed by *in situ* hybridization in neural tube sections of chicken embryos (HH15) electroporated with plasmids encoding for truncated (*ASXL*^*PSC*^) or full-length chicken ASXL1 coupled to GFP. I, injected side; U, control side. n = 3 sections per 2 embryos. Scale bar, 50 μm. (E) Representative bright-field image of chicken embryo electroporated with truncated chicken *ASXL1* transcript as in (C), showing craniofacial malformations (arrow, missing periocular tissue) on the injected side in comparison with the uninjected control (CTRL) side. n = 6 embryos. Scale bars, 200 μm. See also Figure S4.

We next investigated how regulation of *ASXL1* could be implicated in the differentiation outcomes displayed by the BOS models. We noted decreased expression of *ASXL1* in NC progenitors derived from *ASXL1*^*PSC/+*^ and *ASXL1*^*PSC/PSC*^ hESC, and a similar effect for the BOS-iPSC lines, which was however not deemed statistically significant (Figure S4A). Moreover, mutant and wild-type *ASXL1* transcripts were found in NC cultures derived from BOS #1 and #2 lines (Figure S4B), and both alleles were expressed at comparable levels in BOS-iPSC #1 lines (Figure S4C). This argues against degradation of the mutant mRNA. We found that overexpression of wild-type *ASXL1* by a stably integrated PiggyBac vector (Table S1) could not rescue the attachment defect of neurospheres derived from *ASXL1*^*PSC/PSC*^ hESCs, nor did it rescue expression of key NC genes (Figures S4D and S4F). These findings suggest a dominant role of putative truncated ASXL1 variants over the wild-type form. Western blot analysis of early NC cells from all BOS models, using the monoclonal antibody raised against the N-terminal domain of ASXL1, revealed barely detectable levels of a putative truncated variant at 100 kDa and enhanced levels of different variants at 130 and 160 kDa (Figure 4A). Collectively, these results raise the possibility that truncated ASXL1 variants, reduction in *ASXL1* levels, and potentially also misexpression of additional ASXL1 variants (Figures S1I, S1J, and 4A) contributed to the observed NC defects.

To substantiate one of these possibilities, we expressed in hESCs a truncated ASXL1 isoform, which was similar to the variant detected in BOS patient 2 (Table S1). Such ectopic expression led to a nearly complete failure of neurospheres to attach and produce migratory NC-like cells (Figure 4B). Moreover, truncated human as well as chicken *ASXL1* that were injected into the anterior neural region of chicken embryos inhibited the delamination of the electroporated GFP-labeled cells, an effect that was not seen in embryos injected with control plasmid encoding only *eGFP* (Figure 4C). Strikingly, *ZIC1* expression was reduced in neural tube cells expressing truncated chicken *ASXL1* but not in embryos electroporated with full-length chicken *ASXL1* (Figures 4D and S4G). Under the same conditions, expression of *TFAP2A* was unaltered (Figure S4G), indicating a specific perturbation of *ZIC1* expression that was similar to the case of *ASXL1*^*PSC/PSC*^ NC progenitors *in vitro* (Figure S3B).

Finally, we analyzed electroporated embryos at later developmental stages, around HH22–25, to assess craniofacial phenotypes caused by expression of truncated *ASXL1*. This revealed decreased eye size and malformation of periocular tissue on the injected side compared with the uninjected side (Figure 4E). We thus concluded that expression of truncated ASXL1 in NC progenitors could lead to BOS-like phenotypes *in vivo*.

Collectively, in this study we created a human pluripotent stem cell toolset for investigating pathological *ASXL1* mutations present in, or resembling genotypes of, BOS patients. These models enabled us to discover the impaired generation of NC cells, which might be partially caused by a defect in early neuroectoderm specification, and our results support an association with reduced induction of *ZIC1*. However, the question of whether the truncated ASXL1 isoforms by themselves, haploinsufficiency, other isoforms, or combinations of these mechanisms underlie BOS pathogenesis warrants additional studies. A similar question exists with respect to truncated endogenous ASXL1 variants that are associated with leukemia (Abdel-Wahab et al., 2012, Balasubramani et al., 2015, Inoue et al., 2016). Thereby, our findings and models are likely pertinent to hematological malignancies. We moreover linked the truncated isoform to craniofacial dysmorphisms, which have strong association with BOS, and therefore propose ASXL1-mediated perturbation of the NC as a potential cause of characteristic symptoms in BOS. A further medical relevance is apparent from the symptomatic similarity of BOS to Bainbridge-Roper syndrome (BRS), a congenital disorder that is associated with *ASXL3* mutations (Bainbridge et al., 2013), which raises the possibility that BRS is caused by similar mechanisms. The results may also explain the occurrence of Dandy-Walker malformation in BOS patients that could take place due to *ZIC1/ZIC4* repression (Grinberg et al., 2004). Finally, the speech and language learning disabilities in BOS (Hastings et al., 2011) may be linked to the reduced *FOXP2* activation (Figure 3A), as this transcription factor has a primary role in speech and language development (Enard et al., 2002). Overall, the established models could help elucidate *ASXL1*-related pathological mechanisms in additional tissues, including the nervous system and the hematopoietic lineage.

## Experimental Procedures

### Generation of Monoclonal Antibodies

Expression and purification of an N-terminal fragment of ASXL1 (amino acids 1–618), followed by immunization of Lou/c rats was performed as previously described (Feederle et al., 2016, Studier, 2005).

### Generation of Pluripotent Stem Cell Lines and Neural Crest Differentiation

Fibroblasts derived from skin biopsies of healthy donors and BOS patients under oversight by local institutional review boards (Magini et al., 2012) were reprogrammed by modified mRNA and episomal plasmids (Diecke et al., 2015, Kunze et al., 2018), and 500 bp deletions in the *ASXL1* gene were induced in iCas9 HUES9 hESC (*ASXL1*^*+/+*^ control line) (Gonzalez et al., 2014). *ASXL1*^*PSC/PSC*^ and *ASXL1*^*+/+*^ hESC lines were stably integrated with PiggyBac vectors harboring either the truncated *ASXL1* cDNA sequence (N-terminal 2,892 bp; PB*-ASXL1*^*PSC*^), the full-length human *ASXL1* cDNA (4,656 bp; PB*-ASXL1*), the *ZIC1* cDNA sequence (amplified from *ZIC1* human cDNA clone, Biocat; PB*-ZIC1*), or an expression cassette for continuous e*GFP* expression.

Neural crest differentiation was performed as described (Bajpai et al., 2010), and the number of attached neurospheres was quantified at day 7 of the protocol.

### Manipulation of Chicken Embryos

According to German animal care guidelines, no IACUC (Institutional Animal Care and Use Committee) approval was necessary to perform chicken embryo experiments.

Neurospheres obtained at day 5 of NC differentiation were inserted into the developing anterior neural region of chicken embryos (HH10; Hamburger and Hamilton, 1992) and operated embryos were isolated at HH22 and analyzed under a fluorescence stereo microscope. To overexpress transcripts in chicken embryos, the truncated chicken (*Gallus gallus*) *ASXL1* cDNA sequence (Gg-*ASXL1*^*PSC*^; N-terminal 2,445 bp), the truncated human *ASXL1* cDNA sequence (h*ASXL1*^*PSC*^; N-terminal 2,892 bp), or the full-length chicken *ASXL1* cDNA sequence (Gg-*ASXL1*; 4,617 bp) were cloned into expression plasmids and electroporated into the developing brain and neural tube at HH9–10 as described (Rehimi et al., 2016). Manipulated chicken embryos were imaged at HH19 and HH24–25 under a fluorescence stereo microscope. *In situ* hybridization experiments in electroporated chicken embryos were performed as described (Rehimi et al., 2016).

### Transcriptome Analysis (RNA Sequencing)

Total RNA was isolated from day 7 NC progenitor cultures and libraries were prepared using the TruSeq Stranded Total RNA LT Library Prep Kit (Illumina) followed by single-end sequencing. Differential gene expression analysis was performed with the DESeq2 package (Love et al., 2014).

### Statistical Analysis

Values are expressed as means ± standard error of the mean; pairwise comparison was performed using Welch's t test or Wilcoxon’s rank-sum test.

## Author Contributions

F.M. and M.D. designed the study. F.M., E.R., and L.M. performed functional experiments. E.R. and A.P. generated iPSC lines. M.M. performed library preparation and analyzed 3′ RNA sequencing experiments. R.R. and A.R.-I. contributed to the chicken embryo experiments. E.K., R.F., A.F., and A.G. produced monoclonal antibodies. V.R. performed MSC analysis. F.M. and M.D. wrote the manuscript with input from the other authors.

## Accession Numbers

Gene expression data from the RNA sequencing experiments have been deposited in NCBI's Gene Expression Omnibus (GEO) under accession numbers GEO: GSE120200 and GSE127269.

## Supplemental Information

Document S1. Supplemental Experimental Procedures, Figures S1–S4, and Tables S1, S3, and S4

Table S2. Downregulated Genes in *ASXL1* Mutant NC Progenitor Cultures, Related to Figure 3

Document S2. Article plus Supplemental Information
