# Supplementary material for: Pathological ASXL1 Mutations and Protein Variants Impair Neural Crest Development
Source: Stem Cell Reports. 2019 Apr 18;12(5):861–8. doi: 10.1016/j.stemcr.2019.03.006 (PMC6524927; doi:10.1016/j.stemcr.2019.03.006)
Supplement: Document S1. Supplemental Experimental Procedures, Figures S1–S4, and Tables S1, S3, and S4 [file mmc1.pdf]

Stem Cell Reports, Volume *12*

Supplemental Information

Pathological ASXL1 Mutations and Protein Variants Impair Neural Crest Development

Friederike Matheus, Ejona Rusha, Rizwan Rehimi, Lena Molitor, Anna Pertek, Miha Modic, Regina Feederle, Andrew Flatley, Elisabeth Kremmer, Arie Geerlof, Valentyna Rishko, Alvaro Rada-Iglesias, and Micha Drukker

Supplemental Table 1. Human induced pluripotent and embryonic stem cell (hiPSC and hESC) lines used in this study. Related to Figures 1-4 and to Supplemental Experimental Procedures. BOS, Bohring-Opitz syndrome; aa, amino acids

**Name**	**Line**	**Generation** (see **Supplemental Experimental Procedures** for details)	**Genomic *ASXL1* background**	**ASXL1 variants expressed**
BOS-iPSC #1-0 #1-1 #2-0 #2-1	iPSC (#1=f, #2=m)	Reprogramming of BOS patient fibroblasts via modified mRNA(-0) and 4-in-1-mini-intronic plasmids(-1)	heterozygous mutation in *ASXL1* (#1: c.2407_2411del5, #2: c.2893C>T)	wildtype ASXL1 (1541 aa) and putative truncated forms (818 aa in #1, 964 aa in #2)
control iPSC	iPSC	reprogramming of control fibroblasts via mmRNA and 4-in-1-mini-intronic plasmids	wildtype: $ASXL1^{+/+}$	wildtype ASXL1
$ASXL1^{+/+}$ hESC (control)	iCas9 hESC (HUES9)	HUES9 with integrated, tetracycline-inducible Cas9	wildtype: $ASXL1^{+/+}$	wildtype ASXL1
$ASXL1^{PSC/+}$	iCas9 hESC	CRISPR/Cas9 mediated deletion of 476 bp in *ASXL1*, leading to premature STOP codon (PSC)	heterozygous mutation in *ASXL1:* c.2419_2894del476	putative truncated ASXL1 (809 aa) and wildtype ASXL1 (1542 aa)
$ASXL1^{PSC/PSC}$	iCas9 hESC	CRISPR/Cas9 mediated deletion/ inversion of 475-476 bp in *ASXL1*, leading to premature STOP codon (PSC)	clones A, B, C (homozygous): c.2419_2894del476 clone D (compound heterozygous): c.2419_2893inv// c.2419_2893del475	putative truncated ASXL1 (809 aa and/or 824 aa)
GFP-$ASXL1^{+/+}$ *GFP-*$ASXL1^{PSC/PSC}$	iCas9 hESC	stable integration of a constitutively expressed GFP cassette in $ASXL1^{+/+}$ and $ASXL1^{PSC/PSC}$ line	$ASXL1^{+/+}$ (Control) or c.2419_2894del476 ($ASXL1^{PSC/PSC}$)	wildtype ASXL1/putative truncated ASXL1 (809 aa and/or 824 aa)
PB-*ZIC1-*$ASXL1^{+/+}$ PB-*ZIC1-*$ASXL1^{PSC/PSC}$	iCas9 hESC	Stable integration of a tetracycline-inducible Piggybac construct PB-*ZIC1*, bearing *GFP* and the *ZIC1* transcript, in $ASXL1^{PSC/PSC}$ and $ASXL1^{+/+}$ line	$ASXL1^{+/+}$ (Control) or c.2419_2894del476 ($ASXL1^{PSC/PSC}$)	wildtype ASXL1/putative truncated ASXL1 (809 aa and/or 824 aa)
PB-$ASXL1^{PSC}$	iCas9 hESC	Stable integration of a tetracycline-inducible Piggybac construct PB-$ASXL1^{PSC}$, bearing *GFP* and the truncated *ASXL1* transcript (N-terminal 2892 bp) in $ASXL1^{+/+}$ hESC	$ASXL1^{+/+}$; random integration of PB-$ASXL1^{PSC}$	Overexpression of truncated ASXL1 construct (964 aa) and endogenous expression of wildtype ASXL1
PB-*ASXL1* in $ASXL1^{+/+}$ *and* $ASXL1^{PSC/PSC}$	iCas9 hESC	Stable integration of a tetracycline-inducible Piggybac construct PB-*ASXL1*, bearing *GFP* and the wildtype *ASXL1* transcript (4656 bp) in $ASXL1^{+/+}$ and $ASXL1^{PSC/PSC}$ hESC	$ASXL1^{+/+}$ (Control) or c.2419_2894del476 ($ASXL1^{PSC/PSC}$); random integration of PB-*ASXL1*	Overexpression of full-length ASXL1 construct (1541 aa) and endogenous expression of wildtype or putative truncated ASXL1 (809 aa)

Supplemental Table 3. Primers and gRNAs used in this study. Related to Supplemental Experimental Procedures.

Name	Application	Forward sequence	Reverse sequence	Chromosome location
ASXL1 gRNA 1	CRISPR-Cas9: 500 bp deletion	CCATTGTCTGCAGGAACGGT		chr20:32435128
ASXL1 gRNA 2		AGTGAAGTAAGGCTGTCAAG		chr20:32435569
ASXL1-GT	Genotyping PCR	GAGCACCCCTGGAAAGTGTA	TGCTTCAGAGTCTCCGTTGA	chr20:32434755+ 32435685
ASXL1-qPCR	SybrGreen qPCR	GCCACAGGTCAAATGAAGC	GGTCCGAGAGTTGATCAGG	
ASXL1-RT	RT-PCR	TCGCAGACATTAAAGCCCGT	CAGAGGCTGTATCCGTGGA	
ASXL1-seq-F	Sequencing of RT products	GAGCACCCCTGGAAAGTGTA	-	
ASXL1-wildtype allele	SybrGreen qPCR	GAAAGTGATGATGAGGAGCAAGG	ACAGGATCCTTCATAGTGGGA	
ASXL1-mutant allele	SybrGreen qPCR	GGGAAAGTGATGATGAGGAGAC	ACAGGATCCTTCATAGTGGGA	
GAPDH	SybrGreen qPCR	TGCACCACCAACTGCTTAGC	GGCATGGACTGTGGTCATGAG	
OCT4	SybrGreen qPCR	CAATTTGCCAAGCTCCTGAAG	AAAGCGGCAGATGGTCGTT	
SOX2	SybrGreen qPCR	CCTCCGGGACATGATCAGCATGTA	GCAGTGTGCCGTTAATGGCCGTG	
NANOG	SybrGreen qPCR	CCTTCCTCCATGGATCTGCTT	CTTGACCGGGACCTTGTCTTC	
SOX9	SybrGreen qPCR	AGGAAGCTCGCGGACCAGTAC	GGTGGTCCTTCTTGTGCTGCAC	
SOX10	SybrGreen qPCR	ATGAACGCCTTCATGGTGTGGG	CGCTTGTCACTTTCGTTCAGCAG	
PAX3	SybrGreen qPCR	GGCTTTCAACCATCTCATTCCCG	GTTGAGGTCTGTGAACGGTGCT	
SLUG	SybrGreen qPCR	ATCTGCGGCAAGGCGTTTTCCA	GAGCCCTCAGATTTGACCTGTC	
TFAP2A	SybrGreen qPCR	GACCTCTCGATCCACTCCTTAC	GAGACGGCATTGCTGTTGGACT	
GBX2	SybrGreen qPCR	GCGGAGGACGGCAAAGGCTTC	GTCGTCTTCCACCTTTGACTCG	
E-CAD	SybrGreen qPCR	GCCTCCTGAAAAGAGAGTGGAAG	TGGCAGTGTCTCTCCAAATCCG	
N-CAD	SybrGreen qPCR	CCCACACCCTGGAGACATTG	GCCGCTTTAAGGCCCTCA	
ZIC1	SybrGreen qPCR	GATGTGCGACAAGTCCTACACG	TGGAGGATTCGTAGCCAGAGCT	
NR2F1	SybrGreen qPCR	TGCCTCAAAGCCATCGTGCTGT	CAGCAGCAGTTTGCCAAAACGG	
NR2F2	SybrGreen qPCR	TGCACGTTGACTCAGCCGAGTA	AAGCACACTGAGACTTTTCCTGC	

Supplemental Table 4. Antibodies used in this study. Related to Supplemental Experimental Procedures.

Name	Application	Manufacturer	Cat.#
ASXL1 (clone 4F6)	Western Blot	This study	-
ACTIN	Western Blot	Cell Signaling	3700
Oct4	Immunocytochemistry	Cell Signaling	2840 (C30A3)
Sox2	Immunocytochemistry	Cell signaling	2748
Nanog	Immunocytochemistry	Abcam	ab21603
Lin28a	Immunocytochemistry	Cell Signaling	3978 (a177)
TFAP2A	Immunocytochemistry	Santa Cruz	sc-184X
Sox9	Immunocytochemistry	supplied by M. Götz	-
Ki67	Immunocytochemistry	supplied by M. Götz	-
p75NTR	Immunocytochemistry	R&D	MAB367
ZIC1	Western Blot	R&D	AF4978-SP
H3	Western Blot	Abcam	ab1791
CD73-APC (clone AD2)	Flow Cytometry	Miltenyj Biotec (human MSC Phenotyping Kit)	130-095-198
CD90-FITC (clone DG3)			
CD105-PE (clone 43A4E1)			
CD45-PerCP (clone 5B1)			
IgG1-FITC (clone: IS5-21F5)			
mouse IgG1-PE (clone: IS5-21F5)			
mouse IgG1-APC (clone: IS5-21F5)			
IgG1-PerCP (clone: IS5-21F5)			
IgG2a-PerCP (clone S43.10)			

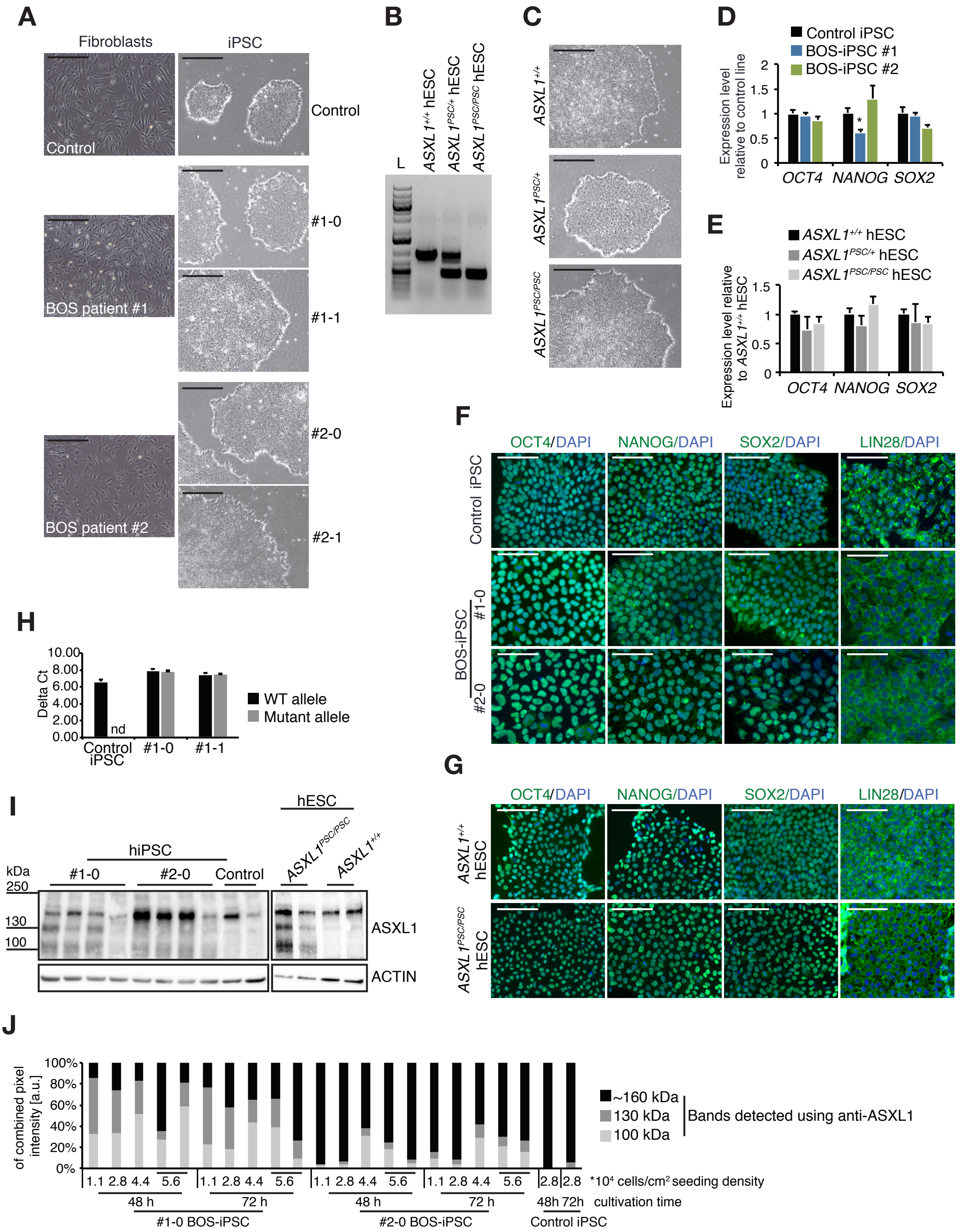

Supplemental Figure S1. Generation of human pluripotent stem cell models for BOS. Related to Figure 1.

Supplemental Figure S1. Generation of human pluripotent stem cell models for BOS. Related to Figure 1. (A) Bright-field images of control and BOS-patient-derived iPSC lines and parental fibroblasts. (B) Analysis of the *ASXL1* locus showing homozygous or heterozygous 476 bp deletion in ASXL1$^{PSC/PSC}$ hESC, clone A, and the ASXL1$^{PSC/+}$ hESC clone, respectively; L, molecular weight marker. (C) Bright-field images of *ASXL1*$^{+/+}$ hESC, *ASXL1*$^{PSC/+}$ and *ASXL1*$^{PSC/PSC}$ modified hESC clones. Overt morphological differences of *ASXL1* mutant to *ASXL1*$^{+/+}$ type were not observed. (D-G) *ASXL1* mutations do not generally affect pluripotency. This was shown by qPCR analysis (D, E) and immunocytochemical analysis (F, G) of core pluripotency transcription factors in undifferentiated BOS-iPSC and control iPSC lines (D, F) and in *ASXL1*^{PSC} hESC lines relative to the *ASXL1*$^{+/+}$ hESC line (E, G). In (D, E), pairwise comparisons between mutant and control lines did not yield statistically significant differences, except for *NANOG* as indicated ($n \geq 3$, different clones/passages). (H) Analysis of mutant and wildtype alleles using primers spanning the 5 bp deletion in BOS iPSC #1 (n=3, different passages, n.d. not detected) confirmed comparable expression levels for both transcripts in BOS-iPSC #1 lines. Samples derived from control iPSCs served as negative control for the detection of the mutant transcript; Ct values were normalized to *GAPDH* to obtain delta Ct values. (I) Extended image of the Blot shown in Fig. 1F, demonstrating detection of several putative protein forms by the monoclonal anti-ASXL1 antibody, which are expressed at varying levels in BOS-iPSC lines and the *ASXL1*$^{PSC/PSC}$ line. (J) Quantification of bands shows variable levels of different protein variants detected using the monoclonal antibody. Analysis of two different Western Blot experiments, one of which is shown in (I), of BOS- and control iPSC lines seeded at different densities and harvested 48 h or 72 h later. For (A) and (C), scale bar indicates 500 μM. In (F) and (G), scale bars represent 200 μM.

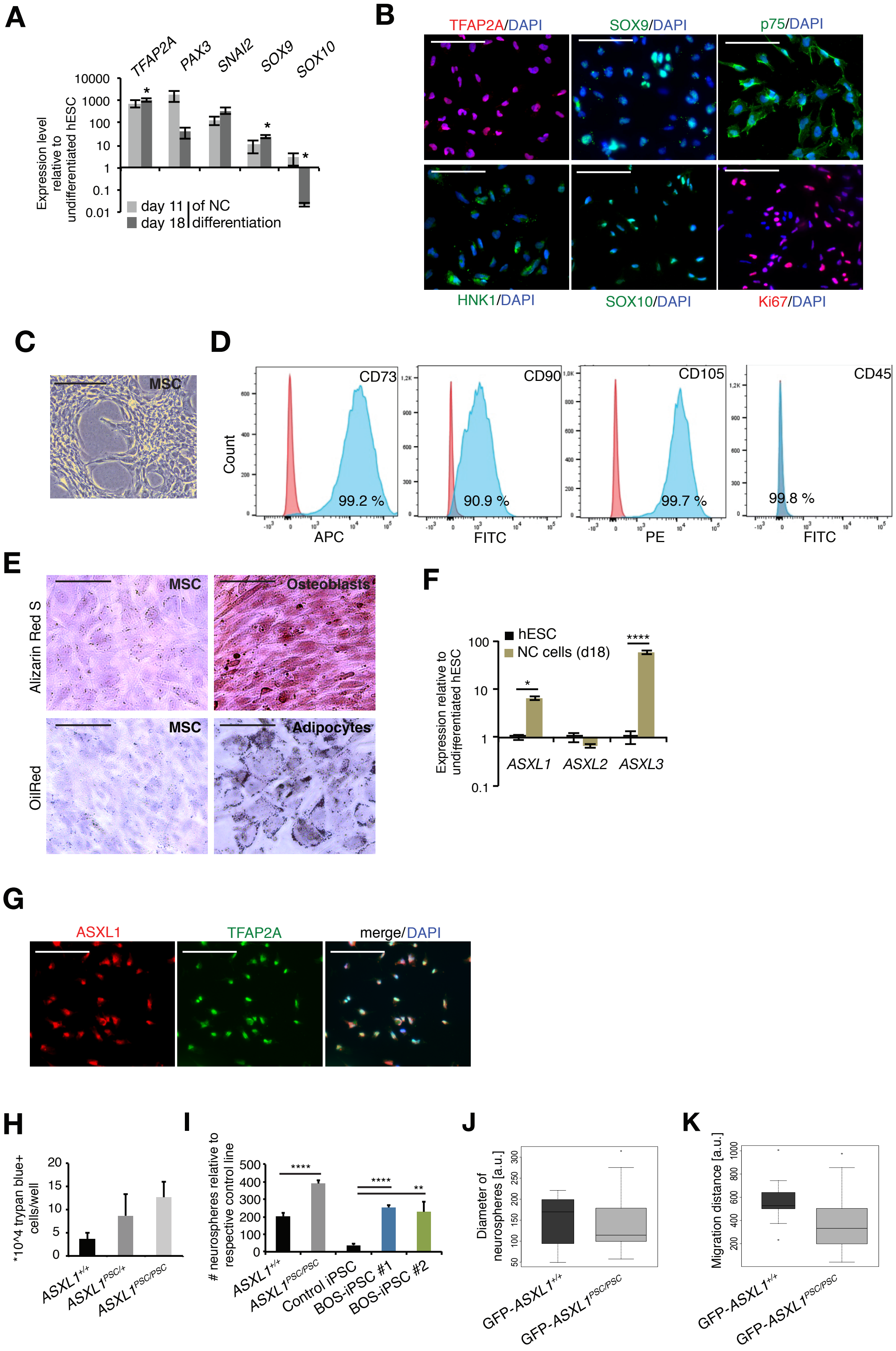

Supplemental Figure S2. Evidence for differentiation of neural crest cells and analysis of the delamination defect in ASXL1 mutant cells. Related to Figure 2.

Supplemental Figure S2. Evidence for differentiation of neural crest cells and analysis of the delamination defect in *ASXL1* mutant cells. Related to Figure 2. (A,B) Analysis of neural crest (NC) markers in $ASXL1^{+/+}$ hESC derived NC cultures by qPCR (A) and immunocytochemistry (B). NC markers including *TFAP2A* and *SNAI2* were highly induced, however pairwise comparison of expression levels in undifferentiated cells and NC cells did not yield significant differences, except for *TFAP2A*, *SOX9* and *SOX10* at day 18 as indicated, due to moderate variation of low transcript levels in undifferentiated cells. Early primitive streak markers *SOX17*, *FOXA2*, *T* (*Brachyury*) and *PAX6* were not detected (not shown; *n*=3 independent experiments). (B) Representative immunocytochemical stainings of NC markers and the proliferation marker Ki67 in passaged NC-like cells (day 18 of differentiation, *n*=3 independent experiments). (C-E) Cells differentiated from NC-like cells by a protocol developed to induce differentiation into mesenchymal stem cells (MSCs) exhibited mesenchymal morphology in bright field microscopy (C), uniformly expressed a cohort of MSC-surface markers but not the hematopoietic marker CD45 as determined by flow cytometry when gates were set by the same IgG control (D), and when exposed to respective differentiation cues, terminally differentiated to osteoblasts and adipocytes as assayed by Alizarin Red and Oil Red O staining, respectively (E; *n*=4 independent experiments). (F) Upregulation of *ASXL1* and *ASXL3* in control hESC-derived NC cells (day 18) relative to undifferentiated hESCs, as demonstrated by qPCR analysis of the *ASXL* genes (n=3 different passages in separate experiments). (G) Representative immunocytochemical staining showing co-localization of ASXL1 and the NC marker TFAP2A in passaged $ASXL1^{+/+}$ hESC-derived NC-like cells (day 18 of differentiation, *n*=3 independent experiments). (H) Quantification of trypan blue positive cells in supernatants of day 7 NC cultures derived from $ASXL1^{+/+}$, $ASXL1^{PSC/+}$ and $ASXL1^{PSC/PSC}$ hESC lines (*n*=3 different clones/passages). (I) Quantification of neurospheres (total numbers including attached and floating neurospheres) derived from $\mathrm{ASXL1}^{PSC/PSC}$ (clones A-D) relative to $ASXL1^{+/+}$ hESCs, and from BOS-iPSC lines relative to control iPSCs, after 7 days of NC differentiation ($n \geq 3$ different clones/passages in separate experiments). (J, K) Analysis of neurospheres based on transplantation experiments in Fig. 2D; $ASXL1^{+/+}$: *n*=9, $ASXL1^{PSC/PSC}$: *n*=21 embryos. (J) Diameter of neurospheres derived from $GFP\text{-}ASXL1^{PSC/PSC}$ and $GFP\text{-} ASXL1^{+/+}$ hESC clones, 48 h following transplantation into chicken embryos; *p*=0.45. (K) Distance between neurospheres and furthest migrated cell, 48 h after transplantation; *p*=0.05. For (B, C, E and G): scale bars indicate 100 μM.

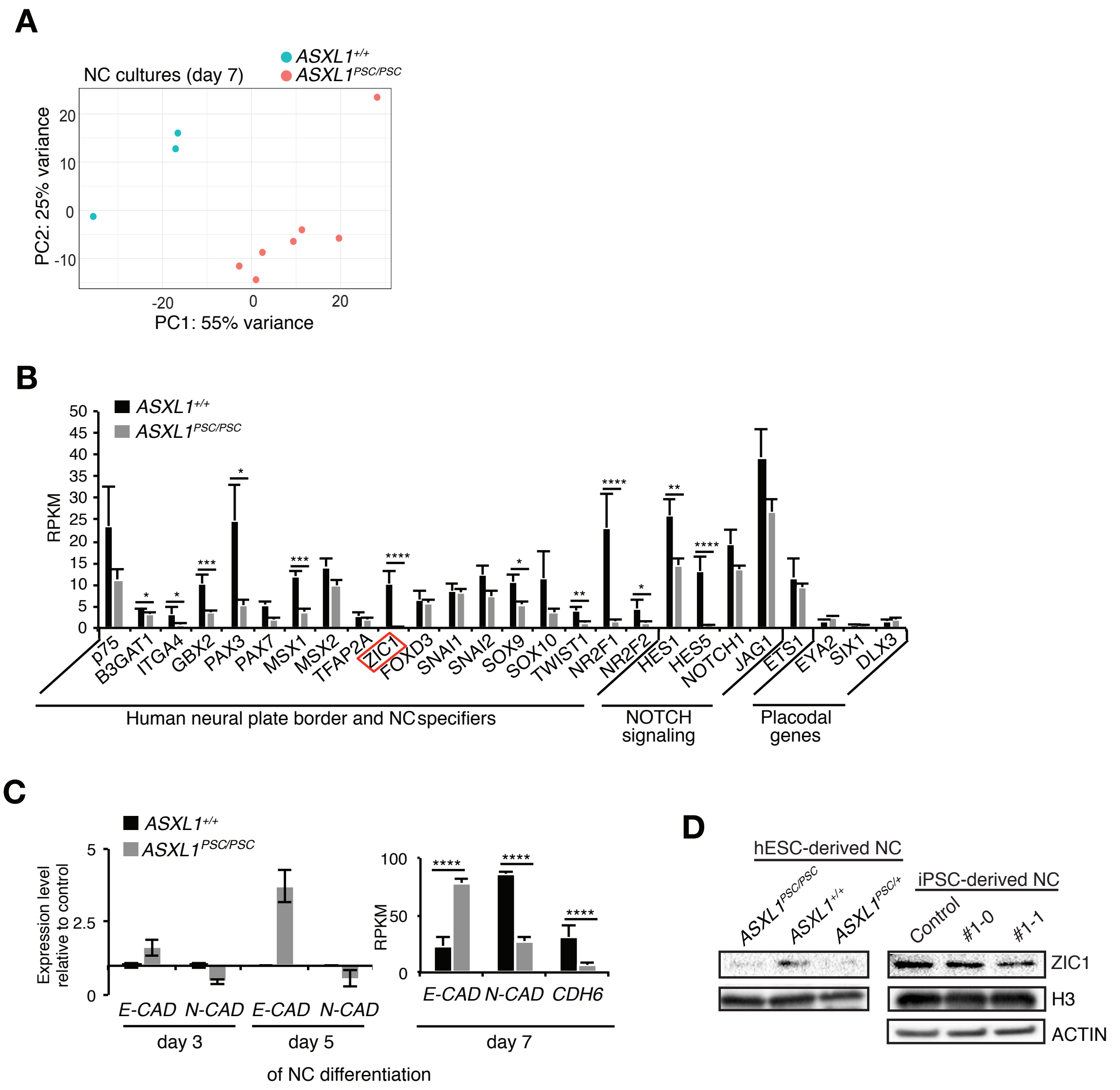

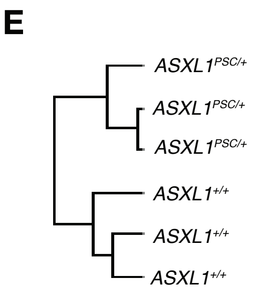

F

Gene	fold change	p(adj)
FOXG1	29,52	0,01920
LHX1	28,50	0,04661
ZIC1	27,89	0,02147
MIXL1	16,53	0,00512
AMN	14,96	0,02282
HYDIN	11,56	0,00191
AC104653.1	9,95	0,02702
TEKT2	8,21	0,00512
MT-TS2	7,98	0,00214
LINC00106	7,55	0,01717

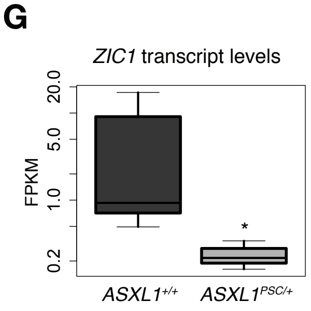

Supplemental Figure S3. Misregulation of NC regulatory networks in *ASXL1*^{PSC} NC progenitors. Related to Fig. 3.

Supplemental Figure S3. Misregulation of NC regulatory networks in *ASXL1*^{PSC} NC progenitors. Related to Fig. 3. (A) Principal component analysis of the global transcriptomes in samples obtained from *ASXL1*$^{PSC/PSC}$ and *ASXL1*$^{+/+}$ hESC line-derived day 7 NC progenitors (*ASXL1*$^{+/+}$: *n*=3; *ASXL1*$^{PSC/PSC}$ clone A and C: *n*=2, *ASXL1*$^{PSC/PSC}$ clone D: *n*=3; based on 3 independent differentiation experiments). (B) Confirmation of NC identity in day 7 progenitor cultures based on expression of published human NC markers and NOTCH signaling pathway members, and low transcript levels of placode-associated genes [based on experiment in (A)]. *ETS1* is a cranial NC cell transcription factor. *ASXL1*$^{PSC/PSC}$-derived cultures showed reduced expression levels of several NC specifiers and NOTCH factors, *ZIC1* was the most significantly downregulated gene. These data highlight the misregulation of NC pathways in *ASXL1*$^{PSC/PSC}$ clones. RPKM, reads per kilobase per million mapped reads. (C) Quantification *of E-Cadherin* (*E-CAD*) and *N-Cadherin* (*N-CAD*) expression at day 3 and 5 (qPCR, *n*=2 different clones/passages), and of *E-CAD, N-CAD* and *Cadherin-6 (CDH6)* expression at day 7 [RNA-seq, based on experiment in (A)] of NC differentiation from *ASXL1*$^{PSC/PSC}$ compared to *ASXL1*$^{+/+}$ cells. Misregulation of *E-CAD* and *N-CAD* in *ASXL1*$^{PSC/PSC}$ cultures suggests perturbation of the Epithelial-to-Mesenchymal Transition (EMT) required for NC delamination. (D). Representative Western Blots for the detection of ZIC1 protein in hESC clones and iPSC-derived NC progenitors at day 7 of differentiation (related to Fig. 3F). H3 controls in hESC-derived samples are the same as for Fig. 4A, as detection of ZIC1 was performed on the same Blots. (E-G) Analysis of global transcriptomes in NC progenitors derived from *ASXL1*$^{PSC/+}$ and *ASXL1*$^{+/+}$ hESC lines confirms general transcriptional deregulation and impaired activation of *ZIC1* in the heterozygous *ASXL1* mutant line. Based on 3'RNA sequencing of samples derived from day 7 NC cultures (*n*=3 different passages for each line, based on samples shown in Fig. 3D). (E) Hierarchical clustering of mapped sequencing reads from *ASXL1*$^{PSC/+}$ and *ASXL1*$^{+/+}$ NC progenitors. (F) The list of topmost downregulated genes in *ASXL1*$^{PSC/+}$ compared to *ASXL1*$^{+/+}$ NC cultures includes *ZIC1* as one of the most strongly reduced transcripts, supporting results obtained in *ASXL1*$^{PSC/PSC}$ NC progenitors. (G) *ZIC1* transcript levels in *ASXL1*$^{PSC/+}$ and *ASXL1*$^{+/+}$ NC progenitors. FPKM, fragments per kilobase per million mapped reads.

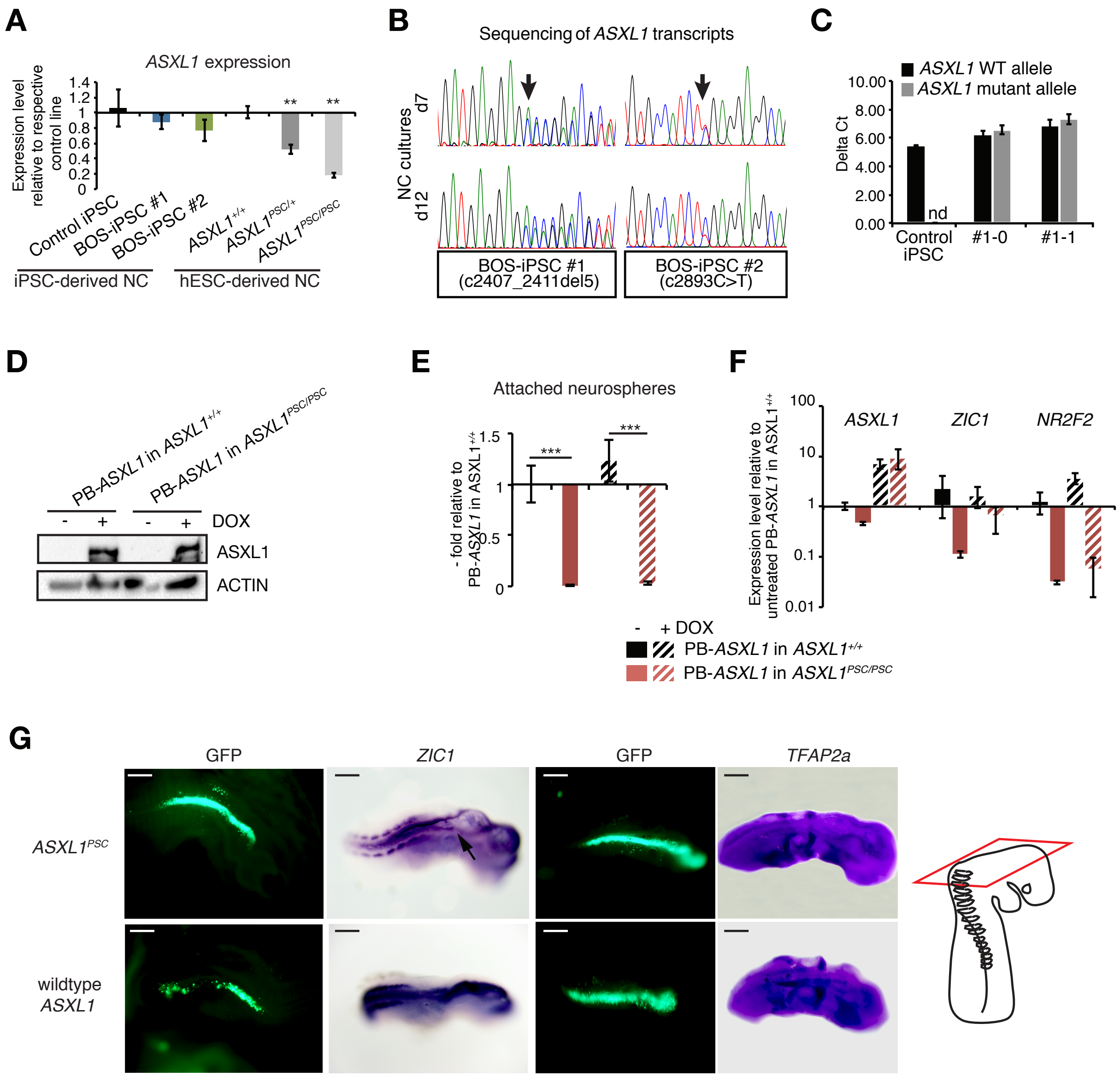

Supplemental Figure S4. Investigation of molecular mechanisms underlying NC development defects in BOS models. Related to Fig. 4.

Supplemental Figure S4. Investigation of molecular mechanisms underlying NC development defects in BOS models. Related to Fig. 4. (A) qPCR analysis of *ASXL1* transcript in day 7 NC cultures derived from BOS-iPSC, $ASXL1^{PSC/+}$ and $ASXL1^{PSC/PSC}$ hESC lines relative to the respective control lines. Primers were specific to Exon 4, thus detecting wildtype and mutant transcripts (*n*=3-6 different clones/passages). (B) Sequences of reverse transcribed *ASXL1* transcripts, isolated from day 7 and day 12 NC cultures of BOS-iPSC #1-0 (day 12), #1-1 (day 7), #2-0 (day 12) and #2-1 (day 7), confirm expression of the mutant transcripts in NC progenitors derived from both patient lines. (C) Discrimination of levels of mutant and wildtype *ASXL1* alleles in samples based on (A), via qPCR, using primers spanning the 5 bp deletion in BOS iPSC #1 (*n*=3 different passages, n.d. not detected). Ct values were normalized against *GAPDH* to obtain delta Ct values. (D-F) Ectopic expression of wildtype *ASXL1* does not rescue the NC differentiation defect in $ASXL1^{PSC/PSC}$ hESC. (D) Western Blot analysis showing the ectopic expression of ASXL1 in DOX-treated $ASXL1^{+/+}$ and $ASXL1^{PSC/PSC}$ cells, harboring stably integrated PB-*ASXL1* expression constructs. (E) Quantification of attached neurospheres with emigrating cells in lines from (D) at day 7 of NC differentiation, left untreated or treated by DOX to ectopically express *ASXL1* from day 1 to day 7 (*n*=10 from 3 independent experiments). Ectopic *ASXL1* expression did not lead to significant improvement of neurosphere attachment in $ASXL1^{PSC/PSC}$ lines. (F) Quantification of *ASXL1, ZIC1* and *NR2F2* expression in lines from (D), with or without DOX treatment from day 1 to day 7 of differentiation. While *ASXL1* transcript levels are comparable in the $ASXL1^{+/+}$ and $ASXL1^{PSC/PSC}$ clones after induction of *ASXL1* expression by DOX treatment, *ZIC1* levels were not entirely rescued, and *NR2F2* levels remained unchanged. *n*=3 different passages; pairwise comparisons between treatments or between genotypes did not yield statistically significant differences due to moderate variations between replicates. (G) Detection of TFAP2A and ZIC1 by *in situ* hybridization in chicken embryos electroporated with plasmids encoding for truncated ($ASXL1^{PSC}$) or wildtype chicken ASXL1 coupled to GFP; related to Fig. 4D. Whole mount images show HH15 embryos as demonstrated by the schematic illustration. The arrow indicates reduced *ZIC1* expression in embryos expressing truncated ASXL1. *n*=2 embryos. Scale bar represents 200 μM.

Supplemental Experimental Procedures

Generation of monoclonal antibodies.

Cloning of construct, protein expression and purification. A 5'fragment of the coding sequence of *ASXL1* (bp 1-1854) was amplified by PCR from human cDNA and cloned into pETM13/LIC containing a C-teminal His_6-tag (supplied by A. Geerlof). The pETM-13/ASXL1 construct was transformed into *E. coli* strain BL21 (DE3) and cultured at 20°C in ZYM 5052 auto-induction medium (Studier, 2005). Cells were harvested after reaching saturation, lysed by sonication, and lysates clarified by centrifugation and filtration (0.2 μM) and applied to a 5 ml HiTrap Chelating HP column (GE Healthcare) using a Äkta Purifier (GE Healthcare). Bound proteins were eluted, fractions containing protein pooled and subsequently applied to size exclusion chromatography using a HiLoad 16/600 Superdex 200 column (GE Healthcare). The fractions containing ASXL1 were pooled and stored at 4°C.

Antibody production. To generate monoclonal antibodies against ASXL1, Lou/c rats were immunized with purified his-tagged human ASXL1 protein (aa 1-618) using standard procedures as described (Feederle et al., 2016). Hybridoma supernatants were validated in enzyme-linked immunoassay (capture and detection ELISA) and by Western blot analysis on control hESC and PB-$ASXL1^{PSC}$ hESC overexpressing truncated ASXL1 protein. The hybridoma cells of ASXL1-reactive supernatants were cloned three times by limiting dilution. Experiments in this study were performed with hybridoma culture supernatant of ASXL1 clones 12F9 and 4F6 (both rat IgG2a/k).

Pluripotent stem cell lines

All iPSC and HUES9 hESC lines were maintained as feeder-free cultures in mTeSR1 medium (STEMCELL Technologies) or StemMACS iPS-Brew XF (Miltenyi Biotec) on Matrigel (Corning)– or Geltrex (Life Technologies)- coated cell culture plates as described (Ludwig et al., 2006) .

Generation of iPSC lines.

mRNA-mediated reprogramming. Bohring-Opitz Syndrome (BOS) fibroblasts were derived from skin biopsies of BOS patients, age 5 and 7 years at the day of procurement [informed consent of the patient's parents given in the original study (Magini et al., 2012)]. Control fibroblasts (ATCC CRL-2522) and BOS fibroblasts were seeded onto NuFF3-RQ cells (GlobalStem, GSC-3404) at day 1; medium was Pluriton Reprogramming Medium (Stemgent) supplemented with 500ng/ml carrier-free B18R Recombinant Protein. From day 3-18, a modified

mRNA cocktail containing *OCT4, SOX2, KLF4, LIN28*, and *C-MYC* mmRNAs at a 3:1:1:1:1 stoichiometric ratio was transfected daily for 4 hours using the Lipofectamine RNAiMAX Transfection Reagent (Thermo Fisher Scientific), followed by washes and addition of fresh reprogramming medium supplemented with B18R. The mmRNA factors were modified with 5-Methyl CTP, Pseudo-UTP, ARCA cap and a 150 nt poly-A tail (provided by the RNA CORE of the Houston Methodist Hospital). Medium was changed to STEMPRO hESC SFM (Thermo Fisher Scientific) from day 16 to day 21, when iPSC colonies were harvested, plated on γ-irradiated mouse embryonic fibroblasts and grown in STEMPRO for 10 additional passages before adapting the iPSCs to a feeder-free culture system.

*Episomal-based reprogramming***.** Control fibroblasts (courtesy of Prof. Magdalena Goetz) and BOS fibroblasts were transfected with 6 or 12 μg of plasmid DNA (MIP 247 CoMiP 4in1 with shRNA p53: pCXLE-hMLN; Addgene #63726 and #27079) using the Nucleofector 2b Device (Lonza) with the MEF 1 Nucleofector Kit (Lonza) and T-020 program. Transfected cells were plated on Matrigel-coated plates and incubated overnight in fibroblast medium supplemented with 10% HyClone Fetal Bovine Serum, 0.2 mM sodium butyrate and 50 μg/mL ascorbic acid. On day 2, medium was changed to Essential 7 medium supplemented with 0.2 mM sodium butyrate and 50 μg/mL ascorbic acid. Between days 15-20, culture conditions were switched to Essential 8 medium and around days 21–30, iPSC colonies were manually selected under the microscope.

Generation of *ASXL1*^{PSC} hESC lines

1x10E6 iCas9 HUES9 hESC (Gonzalez et al., 2014) were electroporated with 3 μg of two gRNA expression plasmids (Supplemental Table 3) using the P3 Primary Cell 4D Nucleofector X Kit (Lonza). Cas9 expression was induced after nucleofection by addition of 1 μg/ml doxycycline for 48h and single cells were plated on Matrigel-coated 15 cm plates in mTeSR1 medium containing 10 μM ROCK-inhibitor (Y-27632; R&D Systems). After 7-10 days, colonies were isolated, passaged, tested for targeted deletion via PCR (for primers see Supplemental Table 3) and Sanger sequencing, and maintained as individual lines.

Generation of hESC lines with stable integration of expression vectors

Generation of lines harboring inducible PB-*ASXL1*^{PSC}, PB-*ASXL1 and* PB-*ZIC1 expression plasmids*

1x10E6 iCas9 HUES9 (Gonzalez et al., 2014) and *ASXL1*$^{PSC/PSC}$ hESC were electroporated as described with a PiggyBac vector containing either the truncated *ASXL1* cDNA sequence (N-terminal 2892 bp), the full-length human ASXL1 cDNA (4656 bp; PB-*ASXL1*), or the *ZIC1* cDNA sequence (amplified from *ZIC1* Human cDNA clone, Biocat), followed by P2A-GFP. The plasmids contained a tetracycline-inducible promoter system and a

Hygromycin resistance gene. A plasmid expressing the Piggybac transposase was co-transfected. Stably integrated clones were selected by 2 weeks' treatment with 50 μg/ml Hygromycin B. To induce overexpression, cells were treated with doxycycline for 24h-48h. Expression of truncated or wildtype ASXL1, or of ZIC1, was tested by qPCR, Western blotting and immunocytochemistry.

Generation of GFP-ASXL1$^{PSC/PSC}$ and GFP-ASXL1$^{+/+}$ hESC lines

ASXL1$^{PSC/PSC}$ and *ASXL1$^{+/+}$* hESC lines were transfected as described above with a transposase-expressing vector together with a PiggyBac vector harboring a *GFP* expression cassette regulated by the CAG promoter. Homogenous cultures of GFP-expressing cells were obtained by sorting using a FACS AriaIII (BD Biosciences) with non-transfected cells serving as a negative control.

Neural crest differentiation and analysis.

*NC differentiation***.** Differentiation to NC was performed essentially as described (Bajpai et al., 2010). Briefly, pluripotent stem cell lines were detached and placed into low attachment plates in NC induction medium to form aggregates (neurospheres). At day 4, neurospheres were plated onto uncoated tissue culture dishes, until NC-like cells had emigrated and could be passaged to dishes coated with 5 μg/ml fibronectin after removal of the neurospheres (around day 11). From then, cells were cultured in maintenance medium, to which 50 pg/ml BMP2 and 3 μM CHIR99021 were added after passage 2. The number of attached, outgrowing neurospheres was quantified at day 7 and related to the total number of floating and attached neurospheres. Attached neurospheres that exhibited uniform emigration of cell sheets or at least 10 NC cells or cell clusters in their direct vicinity (approximately 100 µm apart) were classified as neurospheres with delaminating, migrating NC cells.

MSC differentiation. For conversion to MSCs, NC cells (>passage 2) were cultivated on uncoated dishes in human StemMACS MSC Expansion Media (Miltenyi Biotec) for around 10 days before morphology was assessed by brightfield microscopy. To test for MSC marker expression, NC-derived MSC were dissociated with 0.25% Trypsin-EDTA and stained for positive and negative MSC markers using the human MSC Phenotyping Kit (Miltenyj Biotec). For controls, cells were incubated with the kit's isotype control cocktail. The analysis was performed on a FACS AriaIII.

Terminal differentiation to osteoblasts/adipocytes. NC-derived MSC were cultivated to 90 % confluence in 12- or 24-well plates, subsequently, medium was changed to human StemMACS OsteoDiff Media or human StemMACS AdipoDiff Media (Miltenyi Biotec) to induce osteoblast and adipocyte differentiation, respectively, while control wells were kept in MSC Expansion Media. After 3 weeks, staining of osteoblasts and controls was

performed by 45 min fixation with 10% Formalin, followed by incubation with 20 mg/ml Alizarin Red S and subsequent washes with de-ionized water. Adipocytes and control cells were fixed first with 10 % Formalin for 45 min, then with 60 % 2-propanol for 5 min. Staining was performed by 3 mg/ml Red Oil O solution for 10 min followed by Mayer's Hematoxylin solution for 5 min, with subsequent tap-water washes. Pictures were taken under a brightfield microscope.

Manipulation of chicken embryos

According to German animal care guidelines, no IACUC (Institutional Animal Care and Use Committee) approval was necessary to perform chicken embryo experiments. Fertilized chicken eggs (*Gallus gallus domesticus*) were obtained from a local breeder (LSL Rhein-Main) and incubated at 37°C and 80% humidity in a normal poultry egg incubator. Following microsurgical procedures, the eggs were re-incubated until the embryos reached the desired developmental stages according to the staging system of HH (Hamburger and Hamilton, 1992).

In ovo transplantation of neurospheres

Neurospheres obtained at day 5 of NC differentiation were inserted into the developing anterior neural region of chicken embryos (8-10 somite stage, HH10) and operated eggs were sealed with medical tape and re-incubated until stage HH22, when the embryos were isolated and analyzed under a fluorescence stereo microscope (Olympus SZX 16; details outlined in Supplemental Methods). Distance between transplanted neurospheres and furthest migrated cell, diameter of neurospheres and the total number of migrated cells were determined using the Fiji software (Schindelin et al., 2012).

In ovo electroporation of chicken embryos

The truncated chicken (*Gallus gallus*) *ASXL1* cDNA sequence (Gg-$ASXL1^{PSC}$; N-terminal 2445 bp), the truncated human *ASXL1* cDNA sequence (h$ASXL1^{PSC}$; N-terminal 2892 bp) or the full-length chicken *ASXL1* cDNA sequence (Gg-*ASXL1*; 4617 bp) were cloned into a pCIG vector harboring a *GFP* coding sequence coupled to the *T2A* cleavage signal. The plasmids were mixed with fast green solution (Sigma) at a 2:1 ratio to ease the detection of the injection site and microinjected into the target site of the developing brain and neural tube of HH9-10 chicken embryos. For electroporation, electrodes were placed on each side of the microinjected brain and neural tube, and five square pulses of 35 V within 20 ms width were applied to each embryo using the Intracel TSS20 OVODYNE Electroporator. For control experiments, the vector expressing only GFP or the full-length chicken ASXL1 sequence was electroporated as described above.

Following electroporation, the eggs were sealed with medical tape and re-incubated until the desired developmental stages of chicken embryos (HH19 and HH24-25), to be imaged under a fluorescence stereo microscope.

In situ hybridization in chicken embryos

Chicken embryos were electroporated at stage HH9 with the plasmids described above and fixed at stage HH15. Fixation and subsequent *in situ* hybridization were performed according to the same protocol and using the same *ZIC1* and *TFAP2A* probes as described before (Rehimi et al., 2016).

Gene expression analysis

RT-PCR and qPCR. RNA was extracted using the RNeasy Mini Kit (Qiagen) and reverse transcribed using the SuperScript III kit (Life Technologies). Power SYBR Green PCR Master Mix or Taqman Gene Expression Master Mix (Life Technologies) were used for qPCR on a QuantStudio 12k Flex (Life Technologies). Primers are listed in Supplemental Table 3. Ct values were normalized against the *GAPDH* housekeeping control to obtain delta Ct values, based on which delta delta Ct values and relative expression levels were calculated.

Taq Polymerase (Qiagen) was used in RT-PCR, and products were subjected to Sanger sequencing.

RNA sequencing. RNA from day 7 NC progenitor cultures (1 well of a 6-well plate per replicate; for global RNA-seq: 3 independent differentiation experiments, one clone of *ASXL1*$^{+/+}$ and 3 clones of *ASXL1*$^{PSC/PSC}$, for 3' RNA-seq: 3 different passages of *ASXL1*$^{+/+}$ and *ASXL1*$^{PSC/+}$ clones) was extracted using the RNeasy Mini Kit and quality assessed using an Agilent 2100 Bioanalyzer with the RNA 6000 Pico Kit (Agilent). For global RNA-seq, libraries were prepared using 1 μg of total RNA with the TruSeq Stranded Total RNA LT Library Prep Kit (with Ribo- Zero Human/Mouse/Rat; Illumina) according to the supplied protocol. Libraries were analyzed with the High Sensitivity DNA Kit (Agilent), pooled, diluted 1:10 and subjected to single-end sequencing for 75 cycles on a NextSeq with the NextSeq 500/550 v2 reagent cartridge (Illumina). For 3'RNA-seq, 0.5 μg of RNA was used for library preparation with the QuantSeq 3' mRNA Library Prep Kit FWD for Illumina (Lexogen) according to the manufacturer's specifications. The libraries were sequenced on the Illumina HiSeq2500 platform, producing 51 nt single-end reads.

RNA-seq data analysis and statistics. Quality of the sequencing run was confirmed via QC reports on the Illumina BaseSpace platform and the FastQC tool provided on the Galaxy platform (Afgan et al., 2016). Reads were mapped to the hg19 genome using TopHat (global RNA-seq) or mapped to GRCh38 using STAR (Dobin et al., 2013)(3'RNA-seq). Read count was performed using Cufflinks 2.2.1 (3'RNA-seq data) or, in the case of

global RA-seq, using the featureCounts (v1.5.0) function of the Subread package (Liao et al., 2014) and the hg19 human gene annotation. Differential gene expression analysis was performed with the DESeq2 package (Love et al., 2014) in R v3.3.2; genes with a maximum read count of zero were considered to be non-expressed and thus removed from further analysis. PCA Plots and Volcano Plots were built using the ggplot2, dplyr and ggrepel packages in R, respectively. Hierarchical clustering was performed using the Seqmonk software (v.1.44.0). To characterize misregulated gene sets, the list of significantly downregulated transcripts (padj<0.05) was submitted to the Genomatix Software Suite GeneRanker under *http://www.genomatix.de*.

Immunocytochemistry

Cells were fixed with 4% Formaldehyde for 10-15 min, permeabilized and blocked and subsequently stained with primary antibodies at 4° C overnight. Secondary antibodies were added for 1 hour at room temperature and slides were mounted and analyzed by fluorescence microscopy. Antibodies are listed in Supplemental Table 4.

Western Blotting

Total protein lysates were prepared in RIPA buffer, separated via SDS PAGE and wet-blotted on nitrocellulose membranes, which were blocked for 1 h and treated with primary antibodies overnight at 4°C. Secondary antibodies were added to membranes for 1 h at room temperature, and detection was done on a ChemiDoc XRS using Clarity Western ECL Substrate (Bio-Rad Laboratories). Intensities were normalized to Actin or H3 band intensities to determine relative protein levels. Antibodies are listed in Supplemental Table 4.

Statistical analysis

For pairwise comparison of means, Shapiro-Wilk test was applied to evaluate normal distribution of samples, and if given, Welch's t-test was applied. Otherwise, Wilcoxon Rank-Sum test was used to test for statistical significance. P-values were indicated in figures as follows: ns - $p>0.05$, * $p<0.05$, ** $p<0.01$, *** $p<0.001$, ****$p_{adj}<0.0001$.

Supplemental References

Afgan, E., Baker, D., van den Beek, M., Blankenberg, D., Bouvier, D., Cech, M., Chilton, J., Clements, D., Coraor, N., Eberhard, C., *et al.* (2016). The Galaxy platform for accessible, reproducible and collaborative biomedical analyses: 2016 update. Nucleic acids research *44*, W3-W10.

Bajpai, R., Chen, D.A., Rada-Iglesias, A., Zhang, J., Xiong, Y., Helms, J., Chang, C.P., Zhao, Y., Swigut, T., and Wysocka, J. (2010). CHD7 cooperates with PBAF to control multipotent neural crest formation. Nature *463*, 958-962.

Dobin, A., Davis, C.A., Schlesinger, F., Drenkow, J., Zaleski, C., Jha, S., Batut, P., Chaisson, M., and Gingeras, T.R. (2013). STAR: ultrafast universal RNA-seq aligner. Bioinformatics *29*, 15-21.

Feederle, R., Gerber, J.K., Middleton, A., Northrup, E., Kist, R., Kremmer, E., and Peters, H. (2016). Generation of Pax1/PAX1-Specific Monoclonal Antibodies. Monoclonal antibodies in immunodiagnosis and immunotherapy.

Gonzalez, F., Zhu, Z., Shi, Z.D., Lelli, K., Verma, N., Li, Q.V., and Huangfu, D. (2014). An iCRISPR platform for rapid, multiplexable, and inducible genome editing in human pluripotent stem cells. Cell stem cell *15*, 215-226.

Hamburger, V., and Hamilton, H.L. (1992). A series of normal stages in the development of the chick embryo. 1951. Developmental dynamics : an official publication of the American Association of Anatomists *195*, 231-272.

Liao, Y., Smyth, G.K., and Shi, W. (2014). featureCounts: an efficient general purpose program for assigning sequence reads to genomic features. Bioinformatics *30*, 923-930.

Love, M.I., Huber, W., and Anders, S. (2014). Moderated estimation of fold change and dispersion for RNA-seq data with DESeq2. Genome biology *15*, 550.

Ludwig, T.E., Levenstein, M.E., Jones, J.M., Berggren, W.T., Mitchen, E.R., Frane, J.L., Crandall, L.J., Daigh, C.A., Conard, K.R., Piekarczyk, M.S., *et al.* (2006). Derivation of human embryonic stem cells in defined conditions. Nature biotechnology *24*, 185-187.

Magini, P., Della Monica, M., Uzielli, M.L., Mongelli, P., Scarselli, G., Gambineri, E., Scarano, G., and Seri, M. (2012). Two novel patients with Bohring-Opitz syndrome caused by de novo ASXL1 mutations. American journal of medical genetics Part A *158A*, 917-921.

Rehimi, R., Nikolic, M., Cruz-Molina, S., Tebartz, C., Frommolt, P., Mahabir, E., Clement-Ziza, M., and Rada-Iglesias, A. (2016). Epigenomics-Based Identification of Major Cell Identity Regulators within Heterogeneous Cell Populations. Cell reports *17*, 3062-3076.

Schindelin, J., Arganda-Carreras, I., Frise, E., Kaynig, V., Longair, M., Pietzsch, T., Preibisch, S., Rueden, C., Saalfeld, S., Schmid, B., *et al.* (2012). Fiji: an open-source platform for biological-image analysis. Nat Methods *9*, 676-682.

Studier, F.W. (2005). Protein production by auto-induction in high density shaking cultures. Protein expression and purification *41*, 207-234.
