# Supplementary material for: Pathological ASXL1 Mutations and Protein Variants Impair Neural Crest Development
Source: Stem Cell Reports. 2019 Apr 18;12(5):861–8. doi: 10.1016/j.stemcr.2019.03.006 (PMC6524927; doi:10.1016/j.stemcr.2019.03.006)
Supplement: Document S2. Article plus Supplemental Information [file mmc3.pdf]

Stem Cell Reports
Report
OPEN ACCESS

Pathological ASXL1 Mutations and Protein Variants Impair Neural Crest Development

Friederike Matheus,[1] Ejona Rusha,[2] Rizwan Rehimi,[3] Lena Molitor,[1] Anna Pertek,[2] Miha Modic,[4,5] Regina Feederle,[6] Andrew Flatley,[6] Elisabeth Kremmer,[7] Arie Geerlof,[8] Valentyna Rishko,[1] Alvaro Rada-Iglesias,[3] and Micha Drukker[1,*]
[1]Institute for Stem Cell Research, Helmholtz Zentrum München GmbH, 85764 Neuherberg, Germany
[2]Institute for Stem Cell Research, iPSC Core Facility, Helmholtz Zentrum München GmbH, 85764 Neuherberg, Germany
[3]Center for Molecular Medicine Cologne (CMMC), 50931 Köln, Germany
[4]The Francis Crick Institute, London NW1 1AT, UK
[5]Department for Neuromuscular Diseases, UCL Queen Square Institute of Neurology, London WC1N 3BG, UK
[6]Institute for Diabetes and Obesity, Monoclonal Antibody Core Facility, Helmholtz Zentrum München GmbH, 85764 Neuherberg, Germany
[7]Institute of Molecular Immunology, Helmholtz Zentrum München GmbH, 85764 Neuherberg, Germany
[8]Institute of Structural Biology, Protein Expression and Purification Facility, Helmholtz Zentrum München GmbH, 85764 Neuherberg, Germany
*Correspondence: micha.drukker@helmholtz-muenchen.de
https://doi.org/10.1016/j.stemcr.2019.03.006

SUMMARY

The neural crest (NC) gives rise to a multitude of fetal tissues, and its misregulation is implicated in congenital malformations. Here, we investigated molecular mechanisms pertaining to NC-related symptoms in Bohring-Opitz syndrome (BOS), a developmental disorder linked to mutations in the Polycomb group factor *Additional sex combs-like 1* (*ASXL1*). Genetically edited human pluripotent stem cell lines that were differentiated to NC progenitors and then xenotransplanted into chicken embryos demonstrated an impairment of NC delamination and emigration. Molecular analysis showed that *ASXL1* mutations correlated with reduced activation of the transcription factor *ZIC1* and the NC gene regulatory network. These findings were supported by differentiation experiments using BOS patient-derived induced pluripotent stem cell lines. Expression of truncated ASXL1 isoforms (amino acids 1–900) recapitulated the NC phenotypes *in vitro* and *in ovo*, raising the possibility that truncated ASXL1 variants contribute to BOS pathology. Collectively, we expand the understanding of truncated ASXL1 in BOS and in the human NC.

INTRODUCTION

The neural crest (NC) is an embryonic progenitor population that gives rise to multiple derivatives, including craniofacial cartilage and bones and peripheral neurons, many of which are associated with birth defects such as craniofacial and cardiac malformations (Etchevers et al., 2006; Mayor and Theveneau, 2013; Snider and Mishina, 2014). Animal studies have shown that initiation of NC development at the neural plate border, as well as subsequent delamination, migration, and terminal differentiation of the NC progenitors, are orchestrated by specific gene regulatory networks (GRNs) (Simoes-Costa and Bronner, 2015). Advancing the understanding of these GRNs in human NC development necessitates modeling by pluripotent stem cells, in particular for investigating the epigenetic mechanisms that regulate them (Bajpai et al., 2010). Nevertheless, the involvement of mutations in histone-modifying enzymes in pathological NC development has not yet been studied.

Bohring-Opitz syndrome (BOS) is a severe congenital disorder associated with *de novo* mutations in the *Additional sex combs-like 1* (*ASXL1*) gene, characterized by symptoms that include developmental delay and musculoskeletal and neurological features (Hastings et al., 2011). ASXL1 is a co-factor of the Polycomb repressive complex 2 (PRC2), and loss of function (Abdel-Wahab et al., 2012) and expression of dominantly acting truncated ASXL1 variants (Balasubramani et al., 2015; Guo et al., 2018) have been associated with transcriptional misregulations in hematological disorders. Whether and how these mechanisms relate to the molecular mechanism of BOS is unknown.

Here, we reasoned that craniofacial malformations such as palate anomalies indicate a potential perturbation of NC development in BOS (Hastings et al., 2011). By analyzing a comprehensive set of genetically edited human embryonic stem cell (hESC) and patient induced pluripotent stem cell (iPSC) lines that we differentiated into NC progenitors, we identified transcriptional perturbations associated with defects in NC development, which we propose provide a putative mechanism for the craniofacial and plausibly other symptoms in BOS patients.

RESULTS AND DISCUSSION

Generation of Human Pluripotent Stem Cell Models for BOS

We obtained dermal fibroblasts from two BOS patients who carry monoallelic premature stop codons (PSCs) in the

This is an open access article under the CC BY license (http://creativecommons.org/licenses/by/4.0/).

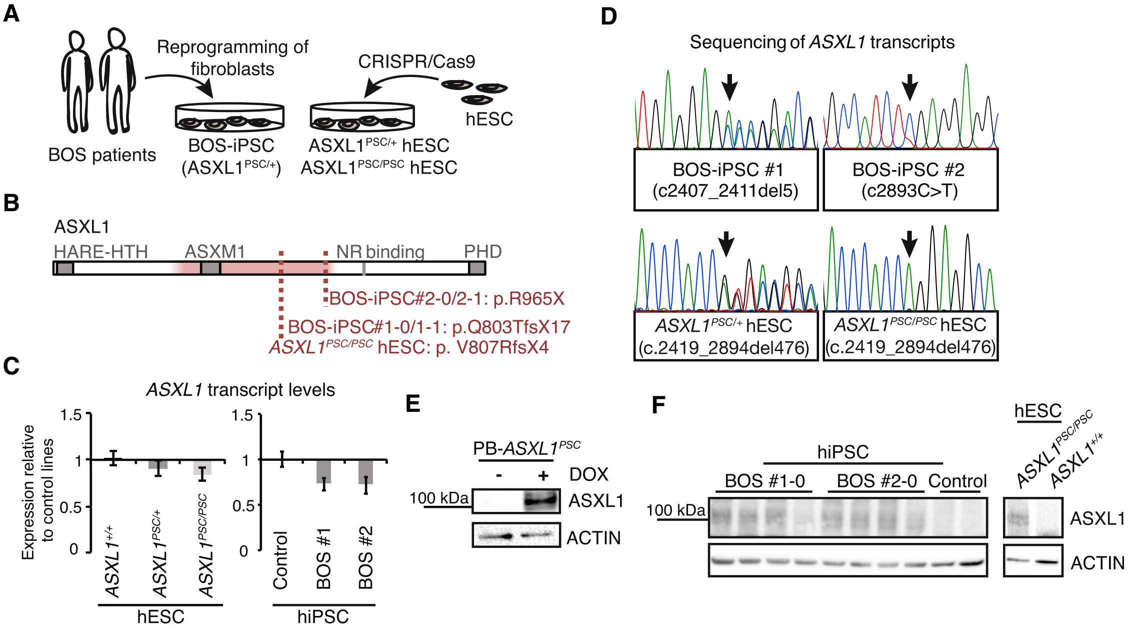

Figure 1. Human Pluripotent Stem Cell Models for Bohring-Opitz Syndrome

(A) Scheme illustrating the generation of human pluripotent stem cell lines carrying premature stop codon (PSC) mutations in *ASXL1*.
(B) Scheme of human *ASXL1* protein showing annotated domains (NR, nuclear receptor), and locations of mutations frequently reported in BOS patients (red tinted sector) and present in BOS-iPSC and *ASXL1*$^{PSC/PSC}$/*ASXL1*$^{PSC/+}$ hESC clones.
(C) Expression of *ASXL1* in BOS-iPSC lines and *ASXL1*$^{PSC/PSC}$/*ASXL1*$^{PSC/+}$ hESC clones relative to the respective iPSC/hESC control lines using primers targeting exon 4 (mean ± SEM, n $\geq$ 3 different clones/passages).
(D) Sequences of reverse transcribed *ASXL1* transcripts from *ASXL1* mutant lines.
(E and F) Representative blotting (n = 3–5 independent experiments) of ASXL1, using a monoclonal antibody raised against the N terminus, in an hESC line overexpressing a truncated ASXL1 variant (PB-*ASXL1*^{PSC}) (E) and in human iPSC and hESC lines (F).
See also Figure S1.

terminal exon of *ASXL1* (Magini et al., 2012). Patient fibroblasts were reprogrammed using two techniques, by episomal plasmids and modified mRNAs, resulting in the derivation of four BOS-iPSC lines (Figure 1A, Table S1, Figure S1A). In addition, we used CRISPR/Cas9 to excise a 500 bp region from the terminal exon of *ASXL1* in hESCs, generating clones that harbor heterozygous and homozygous BOS patient-like PSC mutations (*ASXL1*$^{PSC/+}$ and *ASXL1*$^{PSC/PSC}$; Figure 1A, Table S1, Figures S1B and S1C). BOS-iPSC and edited hESC lines expressed canonical pluripotency markers and displayed colony morphology comparable with control lines, which were the parental *ASXL1*$^{+/+}$ hESC line and two control iPSC lines, respectively (Figure S1A and S1C–S1G, Table S1).

Plausible molecular mechanisms of BOS include *ASXL1* haploinsufficiency and/or expression of truncated ASXL1 (Figure 1B). Testing the level of *ASXL1* transcripts in undifferentiated cells did not reveal a difference between BOS-iPSC, *ASXL1*$^{PSC/+}$, and *ASXL1*$^{PSC/PSC}$ clones and the respective control lines (Figure 1C). We confirmed the presence of mutant *ASXL1* transcripts in the genetically edited hESC clones, and in both BOS-iPSC lines #1-0 and #2-0 by Sanger sequencing (Figure 1D). Furthermore, we detected comparable levels of the normal and mutant transcripts using a primer set specifically targeting the 5-bp deletion in BOS-iPSC lines #1-0/#1-1 (Figure S1H). To further investigate the expression of truncated ASXL1 variants, we raised a monoclonal antibody targeting the N terminus of ASXL1, which by western blotting detected a truncated ASXL1 variant that was overexpressed in undifferentiated hESCs (PB-*ASXL1*^{PSC}; Table S1, Figure 1E). This antibody also detected putative truncated protein isoforms in BOS-iPSC lines #1 and #2, and *ASXL1*$^{PSC/PSC}$ hESC clones (Figures 1F and S1I), albeit at varying levels (Figure S1J). Collectively, this indicates that expression of truncated ASXL1 variants is a possible mode of misregulation in BOS.

ASXL1 Mutations Perturb NC Development

Craniofacial symptoms that are common in BOS patients prompted us to investigate a possible connection to the NC lineage in the hESC and iPSC models. We utilized a differentiation protocol that generated delaminating NC-like cells (Bajpai et al., 2010) from neuroepithelial spheres (neurospheres; Figure 2A), which expressed the characteristic

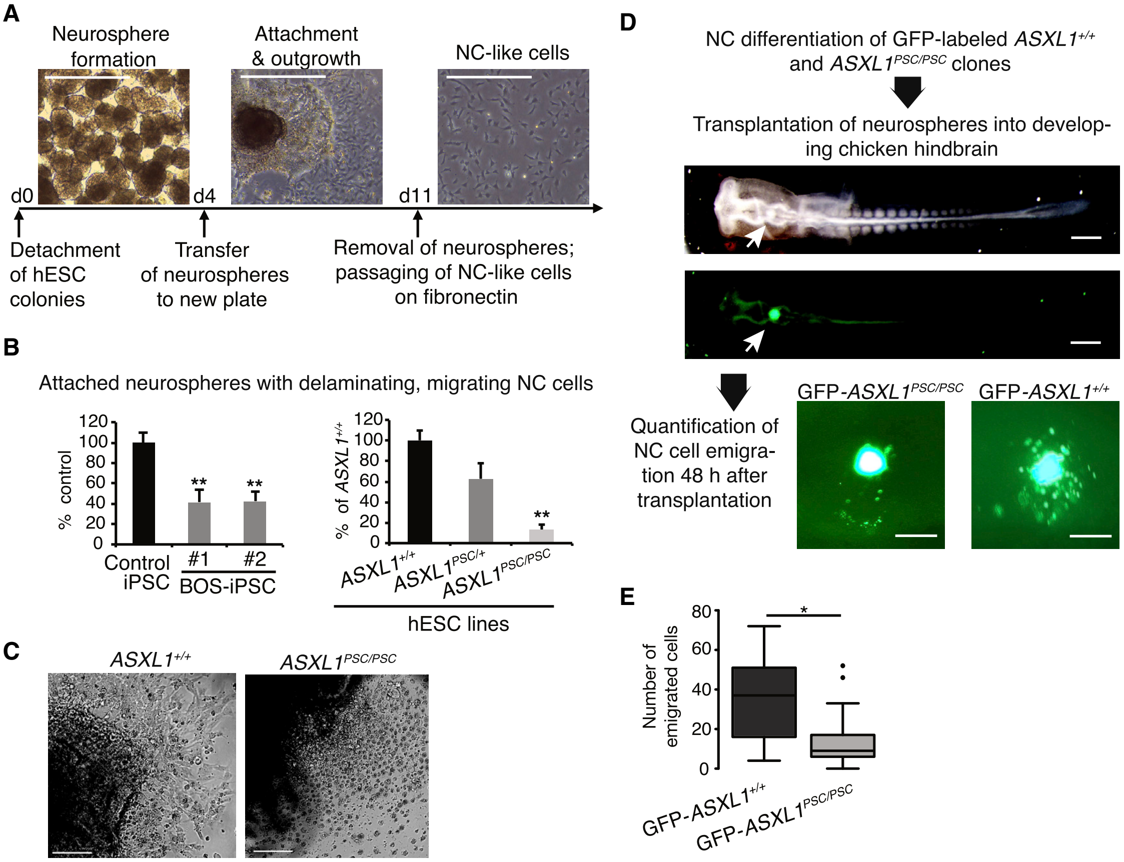

Figure 2. *ASXL1* Mutations Impair Differentiation of Neural Crest Progenitors

(A) Timeline of neural crest (NC) differentiation protocol and bright-field images during *in vitro* differentiation of *ASXL1*$^{+/+}$ hESCs. Scale bar, 500 μm.

(B) Percentage of attached neurospheres with emigrating cells at day 7 of NC differentiation of BOS-iPSC lines and *ASXL1*$^{PSC/PSC}$/*ASXL1*$^{PSC/+}$ clones (mean ± SEM, n = 3–13 different passages/clones and independent experiments, all relative to respective control lines; **p<0.01).

(C) Representative bright-field images of neurospheres at day 7 of NC differentiation. Scale bar, 100 μm.

(D) Workflow of neurosphere transplantation experiments and representative bright-field and GFP images of chicken embryos directly (upper panel) and 48 h (lower panel) after transplantation of neurospheres (white arrows: exemplary transplanted GFP-*ASXL1*$^{+/+}$ neurosphere; dorsal view). Scale bar, 500 μm (chicken embryo) and 200 μm (neurospheres).

(E) Quantification of migrating cells from experiments in (D); *ASXL1*$^{+/+}$, n = 9; *ASXL1*$^{PSC/PSC}$, n = 21 embryos (mean ± SEM, *p = 0.037). Single dots indicate outliers.

See also Figure S2.

NC markers p75NTR, *SOX9*, *SOX10*, *TFAP2A*, *PAX3*, and *SNAI2* (Lee et al., 2007; Simoes-Costa and Bronner, 2015) and the proliferation marker KI67 (Figures S2A and S2B). Moreover, in accordance with the expected potency of NC progenitors, they readily gave rise to putative mesenchymal stem cells (MSCs) (Noden and Trainor, 2005) that exhibited spindle-shaped morphology, expression of consensus MSC surface markers CD73, CD90, and CD105 (Ramos et al., 2016), and terminally differentiated into osteoblasts and adipocytes (Figures S2C–S2E).

We noted upregulation of *ASXL1* and *ASXL3*, but not of *ASXL2*, in NC progenitors (Figure S2F), indicating specific roles of the two genes during NC differentiation, which was further supported by co-expression of ASXL1 and the NC regulator TFAP2A (Figure S2G). Importantly, neurospheres derived from BOS-iPSC lines with heterozygous *ASXL1* mutations displayed reduced attachment and diminished emigration of NC cells in comparison with control iPSC-derived neurospheres (Figure 2B, left panel). In the genetically edited hESC lines, we observed a similar reduction in neurosphere attachment and NC emigration, an effect that was statistically significant only in homozygous *ASXL1*$^{PSC/PSC}$ clones (Figure 2B, right panel), with the presence of dead, floating cells surrounding the

neurospheres (Figures 2C and S2H). *ASXL1* mutations did not negatively affect the propensity to form neurospheres; conversely, an increase in the total number of neurospheres was observed in lines harboring mutant *ASXL1* (Figure S2I). Thus, *ASXL1* mutations perturbed the generation and/or delamination of the NC *in vitro*, potentially via defective induction or specification already at the neurosphere stage.

To further test the emigration of NC cells *in vivo*, we performed orthotopic xenotransplantation of neurospheres obtained from eGFP-labeled $ASXL1^{PSC/PSC}$ and $ASXL^{+/+}$ hESC lines (Table S1) into Hamburger-Hamilton (HH) stage 10 chicken embryos (Figure 2D). Neurospheres derived from $ASXL1^{PSC/PSC}$ hESC clones exhibited a significant reduction in the number of emigrating cells compared with neurospheres derived from the parental $ASXL1^{+/+}$ hESC line (Figures 2D and 2E), an effect that was not due to size differences between control and mutant neurospheres (Figure S2J). Cells that emigrated from the neurospheres of both lines transversed a similar distance (Figure S2K). Collectively, these results indicate a connection between *ASXL1*, NC development, and BOS.

ASXL1 Mutations Impair Activation of *ZIC1* and the NC Gene Regulatory Network

To delineate mechanisms underlying the paucity and migration defect of the NC progenitors, we conducted global RNA sequencing. This revealed mutually exclusive clustering of samples derived from NC progenitors harboring the $ASXL1^{PSC/PSC}$ genotype and the parental control cells (Figure S3A).

Strikingly, the neural plate border transcription factors *ZIC1* and *ZIC4* (Merzdorf, 2007), which are an immediately adjacent gene pair, were among the most negatively regulated transcripts in the $ASXL1^{PSC/PSC}$ NC progenitors (Figure 3A). This substantiated the link to BOS and related syndromes, because heterozygous deletion of the *ZIC1*/*ZIC4* locus is associated with Dandy-Walker malformation (Grinberg et al., 2004), a defect in cerebellar development that is observed in BOS patients (Hastings et al., 2011). Moreover, negatively regulated genes in the dataset were significantly associated with several disorders and malformations found in BOS, including agenesis of the corpus callosum, craniofacial dysmorphisms, musculoskeletal abnormalities, and seizures (Figure 3B).

ZIC1, which was 50-fold reduced in $ASXL1^{PSC/PSC}$ NC cultures, is at the apex of the GRN controlling NC induction (Simoes-Costa and Bronner, 2015). Accordingly, transcription factors that promote further specification, delamination, and migration of the NC were also negatively regulated in the $ASXL1^{PSC/PSC}$ NC cells, including *PAX3*, *GBX2*, *MSX1*, *NR2F1/2*, *ZEB2*, *COL2A1*, *TWIST1*, *SOX5*, Cadherins, and Ephrins and their cognate receptors (Rada-Iglesias et al., 2012; Simoes-Costa and Bronner, 2015) (Figures 3C, S3B, and S3C, Table S2). qPCR, performed to confirm the transcriptional changes for a subset of NC genes, demonstrated significant downregulation in the $ASXL1^{PSC/PSC}$ clones and mild reduction in the patient lines (Figures 3D and 3E), and ZIC1 protein levels were significantly lower in NC cultures derived from one BOS-iPSC line (Figures 3F and S3D). To further analyze the connection of the $ASXL1^{PSC/+}$ ESC line to NC defects, we performed 3′ mRNA sequencing of NC cultures derived from the heterozygous $ASXL^{PSC/+}$ clones and control hESCs. This revealed mutually exclusive clustering of the two genotypes and a significant 28-fold downregulation of *ZIC1* in $ASXL^{PSC/+}$ NC progenitors ($p_{adj} < 0.05$; Figures S3E–S3G). Collectively, this supports a link between BOS-associated mutations in *ASXL1* and *ZIC1* misregulation.

To investigate whether reduced *ZIC1* levels could indeed underlie the observed NC differentiation phenotypes, we stably integrated an inducible *ZIC1* transgene in $ASXL1^{+/+}$ and $ASXL1^{PSC/PSC}$ hESC clones (Figure 3G, Table S1). Strikingly, overexpression of *ZIC1* during NC differentiation restored the attachment and delamination phenotype of NC-like cells from $ASXL1^{PSC/PSC}$ neurospheres (Figure 3H). These results collectively indicate that impairment of ZIC1 activation contributes to the observed differentiation defects when *ASXL1* is mutated.

Expression of Truncated ASXL1 Recapitulates NC Developmental Effects

We next investigated how regulation of *ASXL1* could be implicated in the differentiation outcomes displayed by the BOS models. We noted decreased expression of *ASXL1* in NC progenitors derived from $ASXL1^{PSC/+}$ and $ASXL1^{PSC/PSC}$ hESC, and a similar effect for the BOS-iPSC lines, which was however not deemed statistically significant (Figure S4A). Moreover, mutant and wild-type *ASXL1* transcripts were found in NC cultures derived from BOS #1 and #2 lines (Figure S4B), and both alleles were expressed at comparable levels in BOS-iPSC #1 lines (Figure S4C). This argues against degradation of the mutant mRNA. We found that overexpression of wild-type *ASXL1* by a stably integrated PiggyBac vector (Table S1) could not rescue the attachment defect of neurospheres derived from $ASXL1^{PSC/PSC}$ hESCs, nor did it rescue expression of key NC genes (Figures S4D and S4F). These findings suggest a dominant role of putative truncated ASXL1 variants over the wild-type form. Western blot analysis of early NC cells from all BOS models, using the monoclonal antibody raised against the N-terminal domain of ASXL1, revealed barely detectable levels of a putative truncated variant at 100 kDa and enhanced levels of different variants at 130 and 160 kDa (Figure 4A). Collectively, these results raise

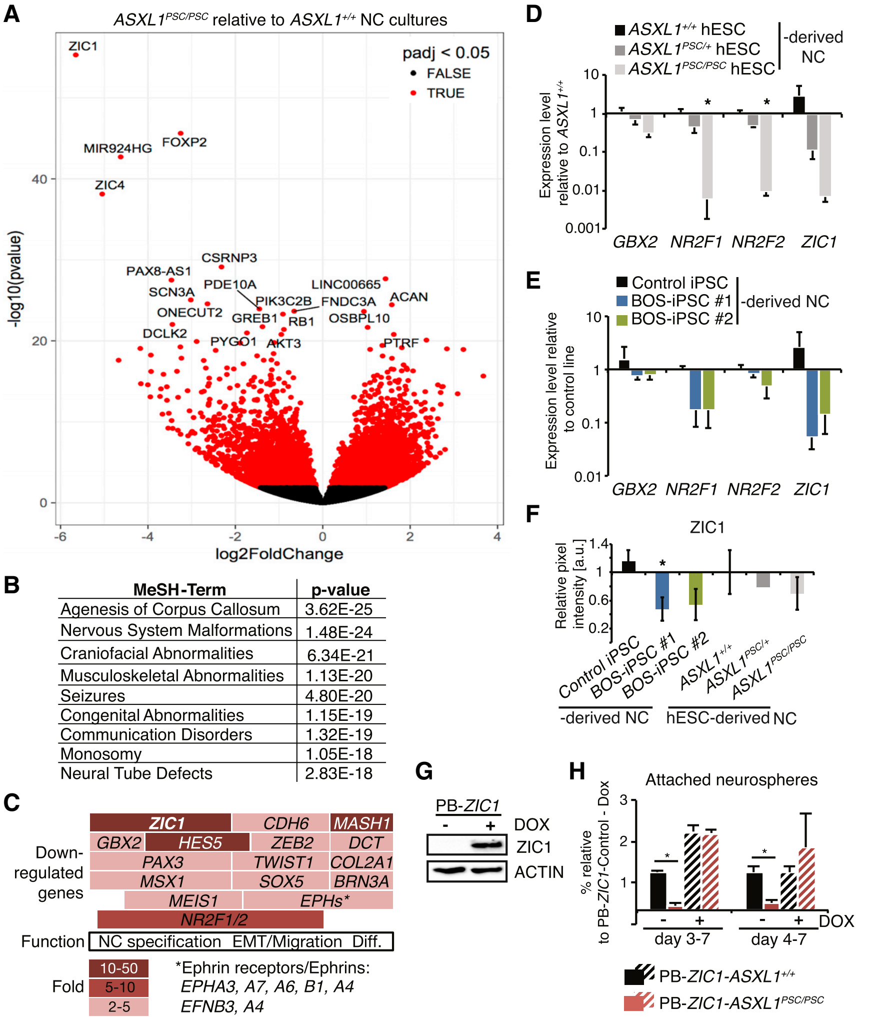

Figure 3. Misregulation of Gene Networks Associated with NC Development and BOS Symptoms in *ASXL1* Mutant Lines

(A) Volcano plot exhibiting differentially expressed genes in *ASXL1*$^{PSC/PSC}$ compared with *ASXL1*$^{+/+}$ day 7 NC progenitors (total mRNA sequencing; n = 3 *ASXL1*$^{+/+}$ and n = 7 *ASXL1*$^{PSC/PSC}$ samples, different clones and independent differentiation experiments).

(B) Most significant Medical Subject Headings (MeSH) terms associated with down-regulated gene sets ($p_{adj} < 0.05$) in (A).

(C) Diagram of genes with annotated functions in NC development and their degree of misregulation ($p_{adj} < 0.05$), based on (A). Diff., terminal differentiation.

(D and E) Relative expression levels of canonical NC genes in day 7 NC differentiation cultures of *ASXL1*^{PSC} hESC lines (D) and BOS-iPSC lines (E) compared with respective control lines (mean ± SEM, n = 3–6 different clones/passages; *NR2F1* in iPSC control, n = 2 different passages; *p < 0.05).

(F) Quantification of ZIC1 protein levels in NC cultures (day 7) derived from cell lines as in (D and E) compared with the respective control lines (based on western blotting, n = 3–4 different clones/passages, mean ± SEM, *p < 0.05).

(G and H) Rescue of the NC differentiation defect by ectopic expression of *ZIC1*. (G) Detection of ZIC1 overexpression in DOX-treated PB-*ZIC1-ASXL1*$^{+/+}$ by western blotting. (H) Analysis of attached neurospheres with emigrating cells in PB-*ZIC1-ASXL1*$^{+/+}$ and PB-*ZIC1-ASXL1*$^{PSC/PSC}$ lines at day 7, −/+ DOX treatment to overexpress *ZIC1* starting at day 3 (n = 2 and 3 independent experiments) or day 4 (n = 3 independent experiments). All data shown as mean ± SEM, *p < 0.05.

See also Figure S3.

the possibility that truncated ASXL1 variants, reduction in *ASXL1* levels, and potentially also misexpression of additional ASXL1 variants (Figures S1I, S1J, and 4A) contributed to the observed NC defects.

To substantiate one of these possibilities, we expressed in hESCs a truncated ASXL1 isoform, which was similar to the variant detected in BOS patient 2 (Table S1). Such ectopic expression led to a nearly complete failure of neurospheres to attach and produce migratory NC-like cells (Figure 4B). Moreover, truncated human as well as chicken *ASXL1* that were injected into the anterior neural region of chicken embryos inhibited the delamination of the electroporated GFP-labeled cells, an effect that was not seen in embryos injected with control plasmid encoding only *eGFP* (Figure 4C). Strikingly, *ZIC1* expression was reduced in neural tube cells expressing truncated chicken *ASXL1* but not in embryos electroporated with full-length chicken *ASXL1* (Figures 4D and S4G). Under the same conditions, expression of *TFAP2A* was unaltered (Figure S4G), indicating a specific perturbation of *ZIC1* expression that was similar to the case of *ASXL1*$^{PSC/PSC}$ NC progenitors *in vitro* (Figure S3B).

Finally, we analyzed electroporated embryos at later developmental stages, around HH22–25, to assess craniofacial phenotypes caused by expression of truncated *ASXL1*. This revealed decreased eye size and malformation of periocular tissue on the injected side compared with the uninjected side (Figure 4E). We thus concluded that expression of truncated ASXL1 in NC progenitors could lead to BOS-like phenotypes *in vivo*.

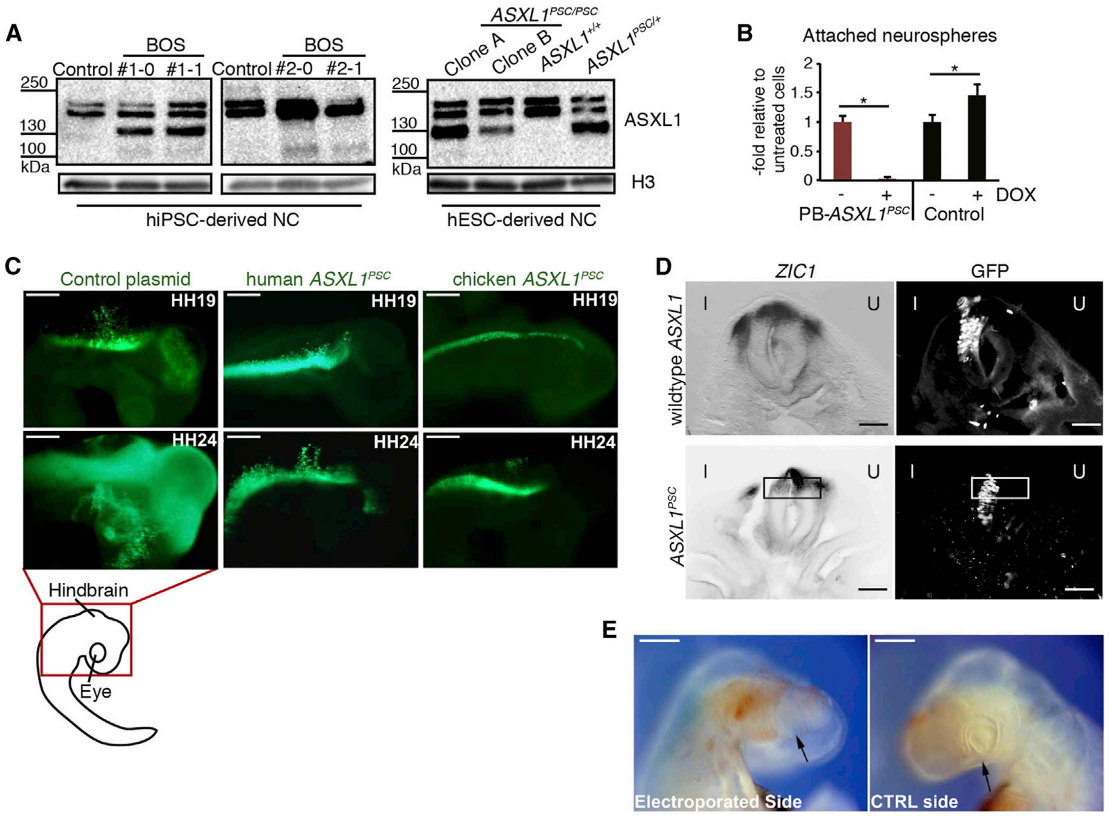

Figure 4. Expression of Truncated ASXL1 Impairs NC Cell Emigration and *ZIC1* Expression

(A) Detection of ASXL1 by western blotting in samples of day 7 NC cultures (monoclonal antibody as in Figures 1E and 1F).

(B) Percentage of attached neurospheres with emigrating cells derived at day 7 from DOX-treated $ASXL1^{+/+}$ hESCs and PB-$ASXL1^{PSC}$ hESCs, overexpressing truncated ASXL1, relative to untreated samples (mean ± SEM, n = 3 independent experiments, $^{*}p < 0.05$).

(C) Representative fluorescent images of chicken embryos 48 h after electroporation with plasmids expressing GFP or GFP coupled to truncated chicken or human $ASXL1^{PSC}$ (n = 10 embryos each); the red rectangle indicates displayed head/hindbrain region. Scale bars, 200 μm.

(D) Expression pattern of *ZIC1* analyzed by *in situ* hybridization in neural tube sections of chicken embryos (HH15) electroporated with plasmids encoding for truncated ($ASXL^{PSC}$) or full-length chicken ASXL1 coupled to GFP. I, injected side; U, control side. n = 3 sections per 2 embryos. Scale bar, 50 μm.

(E) Representative bright-field image of chicken embryo electroporated with truncated chicken *ASXL1* transcript as in (C), showing craniofacial malformations (arrow, missing periocular tissue) on the injected side in comparison with the uninjected control (CTRL) side. n = 6 embryos. Scale bars, 200 μm.

See also Figure S4.

Collectively, in this study we created a human pluripotent stem cell toolset for investigating pathological *ASXL1* mutations present in, or resembling genotypes of, BOS patients. These models enabled us to discover the impaired generation of NC cells, which might be partially caused by a defect in early neuroectoderm specification, and our results support an association with reduced induction of *ZIC1*. However, the question of whether the truncated ASXL1 isoforms by themselves, haploinsufficiency, other isoforms, or combinations of these mechanisms underlie BOS pathogenesis warrants additional studies. A similar question exists with respect to truncated endogenous ASXL1 variants that are associated with leukemia (Abdel-Wahab et al., 2012; Balasubramani et al., 2015; Inoue et al., 2016). Thereby, our findings and models are likely pertinent to hematological malignancies. We moreover linked the truncated isoform to craniofacial dysmorphisms, which have strong association with BOS, and therefore propose ASXL1-mediated perturbation of the NC as a potential cause of characteristic symptoms in BOS. A further medical relevance is apparent from the symptomatic similarity of BOS to Bainbridge-Roper syndrome (BRS), a congenital disorder that is associated with *ASXL3* mutations (Bainbridge et al., 2013), which raises

the possibility that BRS is caused by similar mechanisms. The results may also explain the occurrence of Dandy-Walker malformation in BOS patients that could take place due to *ZIC1/ZIC4* repression (Grinberg et al., 2004). Finally, the speech and language learning disabilities in BOS (Hastings et al., 2011) may be linked to the reduced *FOXP2* activation (Figure 3A), as this transcription factor has a primary role in speech and language development (Enard et al., 2002). Overall, the established models could help elucidate *ASXL1*-related pathological mechanisms in additional tissues, including the nervous system and the hematopoietic lineage.

EXPERIMENTAL PROCEDURES

Generation of Monoclonal Antibodies

Expression and purification of an N-terminal fragment of ASXL1 (amino acids 1–618), followed by immunization of Lou/c rats was performed as previously described (Feederle et al., 2016; Studier, 2005).

Generation of Pluripotent Stem Cell Lines and Neural Crest Differentiation

Fibroblasts derived from skin biopsies of healthy donors and BOS patients under oversight by local institutional review boards (Magini et al., 2012) were reprogrammed by modified mRNA and episomal plasmids (Diecke et al., 2015; Kunze et al., 2018), and 500 bp deletions in the *ASXL1* gene were induced in iCas9 HUES9 hESC ($ASXL1^{+/+}$ control line) (Gonzalez et al., 2014). $ASXL1^{PSC/PSC}$ and $ASXL1^{+/+}$ hESC lines were stably integrated with PiggyBac vectors harboring either the truncated *ASXL1* cDNA sequence (N-terminal 2,892 bp; PB-$ASXL1^{PSC}$), the full-length human *ASXL1* cDNA (4,656 bp; PB-*ASXL1*), the *ZIC1* cDNA sequence (amplified from *ZIC1* human cDNA clone, Biocat; PB-*ZIC1*), or an expression cassette for continuous e*GFP* expression.

Neural crest differentiation was performed as described (Bajpai et al., 2010), and the number of attached neurospheres was quantified at day 7 of the protocol.

Manipulation of Chicken Embryos

According to German animal care guidelines, no IACUC (Institutional Animal Care and Use Committee) approval was necessary to perform chicken embryo experiments.

Neurospheres obtained at day 5 of NC differentiation were inserted into the developing anterior neural region of chicken embryos (HH10; Hamburger and Hamilton, 1992) and operated embryos were isolated at HH22 and analyzed under a fluorescence stereo microscope. To overexpress transcripts in chicken embryos, the truncated chicken (*Gallus gallus*) *ASXL1* cDNA sequence (Gg-$ASXL1^{PSC}$; N-terminal 2,445 bp), the truncated human *ASXL1* cDNA sequence (h$ASXL1^{PSC}$; N-terminal 2,892 bp), or the full-length chicken *ASXL1* cDNA sequence (Gg-*ASXL1*; 4,617 bp) were cloned into expression plasmids and electroporated into the developing brain and neural tube at HH9–10 as described (Rehimi et al., 2016). Manipulated chicken embryos were imaged at HH19 and HH24–25 under a fluorescence stereo microscope. *In situ* hybridization experiments in electroporated chicken embryos were performed as described (Rehimi et al., 2016).

Transcriptome Analysis (RNA Sequencing)

Total RNA was isolated from day 7 NC progenitor cultures and libraries were prepared using the TruSeq Stranded Total RNA LT Library Prep Kit (Illumina) followed by single-end sequencing. Differential gene expression analysis was performed with the DESeq2 package (Love et al., 2014).

Statistical Analysis

Values are expressed as means ± standard error of the mean; pairwise comparison was performed using Welch's t test or Wilcoxon's rank-sum test.

ACCESSION NUMBERS

Gene expression data from the RNA sequencing experiments have been deposited in NCBI's Gene Expression Omnibus (GEO) under accession numbers GEO: GSE120200 and GSE127269.

SUPPLEMENTAL INFORMATION

Supplemental Information can be found online at https://doi.org/10.1016/j.stemcr.2019.03.006.

AUTHOR CONTRIBUTIONS

F.M. and M.D. designed the study. F.M., E.R., and L.M. performed functional experiments. E.R. and A.P. generated iPSC lines. M.M. performed library preparation and analyzed 3′ RNA sequencing experiments. R.R. and A.R.-I. contributed to the chicken embryo experiments. E.K., R.F., A.F., and A.G. produced monoclonal antibodies. V.R. performed MSC analysis. F.M. and M.D. wrote the manuscript with input from the other authors.

ACKNOWLEDGMENTS

We thank the patients' families and following scientists for generously providing fibroblasts: Pamela Magini (S. Orsola-Malpighi Hospital, University of Bologna, Bologna, Italy), Matteo Della Monica (Meyer Children's Hospital, University of Florence, Florence, Italy), Gioacchino Scarano (Medical Genetics Unit, A.O.R.N "G. Rummo", Benevento, Italy), and Prof. Maria Luisa Giovannucci Uzielli (Genetics and Molecular Medicine, University of Florence, Florence, Italy). We would also like to thank Meino Rohlfs and Jens Hinke (Klinikum LMU, Munich) and Christine Wurmser (TU Munich) for providing help with RNA sequencing, Lukas Simon (Helmholtz Zentrum München, Neuherberg) for support with bioinformatics data analysis, and Dmitry Shaposhnikov (Helmholtz Zentrum München) for providing plasmids.

Received: June 11, 2018
Revised: March 18, 2019
Accepted: March 19, 2019
Published: April 18, 2019

REFERENCES

Abdel-Wahab, O., Adli, M., LaFave, L.M., Gao, J., Hricik, T., Shih, A.H., Pandey, S., Patel, J.P., Chung, Y.R., Koche, R., et al. (2012). ASXL1 mutations promote myeloid transformation through loss of PRC2-mediated gene repression. Cancer Cell *22*, 180–193.

Bainbridge, M.N., Hu, H., Muzny, D.M., Musante, L., Lupski, J.R., Graham, B.H., Chen, W., Gripp, K.W., Jenny, K., Wienker, T.F., et al. (2013). De novo truncating mutations in ASXL3 are associated with a novel clinical phenotype with similarities to Bohring-Opitz syndrome. Genome Med. *5*, 11.

Bajpai, R., Chen, D.A., Rada-Iglesias, A., Zhang, J., Xiong, Y., Helms, J., Chang, C.P., Zhao, Y., Swigut, T., and Wysocka, J. (2010). CHD7 cooperates with PBAF to control multipotent neural crest formation. Nature *463*, 958–962.

Balasubramani, A., Larjo, A., Bassein, J.A., Chang, X., Hastie, R.B., Togher, S.M., Lahdesmaki, H., and Rao, A. (2015). Cancer-associated ASXL1 mutations may act as gain-of-function mutations of the ASXL1-BAP1 complex. Nat. Commun. *6*, 7307.

Diecke, S., Lu, J., Lee, J., Termglinchan, V., Kooreman, N.G., Burridge, P.W., Ebert, A.D., Churko, J.M., Sharma, A., Kay, M.A., et al. (2015). Novel codon-optimized mini-intronic plasmid for efficient, inexpensive, and xeno-free induction of pluripotency. Sci. Rep. *5*, 8081.

Enard, W., Przeworski, M., Fisher, S.E., Lai, C.S., Wiebe, V., Kitano, T., Monaco, A.P., and Paabo, S. (2002). Molecular evolution of FOXP2, a gene involved in speech and language. Nature *418*, 869–872.

Etchevers, H.C., Amiel, J., and Lyonnet, S. (2006). Molecular bases of human neurocristopathies. Adv. Exp. Med. Biol. *589*, 213–234.

Feederle, R., Gerber, J.K., Middleton, A., Northrup, E., Kist, R., Kremmer, E., and Peters, H. (2016). Generation of Pax1/PAX1-specific monoclonal antibodies. Monoclonal antibodies in immunodiagnosis and immunotherapy. Monoclon. Antib. Immunodiagn. Immunother. *35*, 259–262.

Gonzalez, F., Zhu, Z., Shi, Z.D., Lelli, K., Verma, N., Li, Q.V., and Huangfu, D. (2014). An iCRISPR platform for rapid, multiplexable, and inducible genome editing in human pluripotent stem cells. Cell Stem Cell *15*, 215–226.

Grinberg, I., Northrup, H., Ardinger, H., Prasad, C., Dobyns, W.B., and Millen, K.J. (2004). Heterozygous deletion of the linked genes ZIC1 and ZIC4 is involved in Dandy-Walker malformation. Nat. Genet. *36*, 1053–1055.

Guo, Y., Yang, H., Chen, S., Zhang, P., Li, R., Nimer, S.D., Harbour, J.W., Xu, M.J., and Yang, F.C. (2018). Reduced BAP1 activity prevents ASXL1 truncation-driven myeloid malignancy in vivo. Leukemia *32*, 1834–1837.

Hamburger, V., and Hamilton, H.L. (1992). A series of normal stages in the development of the chick embryo. 1951. Dev. Dyn. *195*, 231–272.

Hastings, R., Cobben, J.M., Gillessen-Kaesbach, G., Goodship, J., Hove, H., Kjaergaard, S., Kemp, H., Kingston, H., Lunt, P., Mansour, S., et al. (2011). Bohring-Opitz (Oberklaid-Danks) syndrome: clinical study, review of the literature, and discussion of possible pathogenesis. Eur. J. Hum. Genet. *19*, 513–519.

Inoue, D., Matsumoto, M., Nagase, R., Saika, M., Fujino, T., Nakayama, K.I., and Kitamura, T. (2016). Truncation mutants of ASXL1 observed in myeloid malignancies are expressed at detectable protein levels. Exp. Hematol. *44*, 172–176.e1.

Kunze, C., Borner, K., Kienle, E., Orschmann, T., Rusha, E., Schneider, M., Radivojkov-Blagojevic, M., Drukker, M., Desbordes, S., Grimm, D., et al. (2018). Synthetic AAV/CRISPR vectors for blocking HIV-1 expression in persistently infected astrocytes. Glia *66*, 413–427.

Lee, G., Kim, H., Elkabetz, Y., Al Shamy, G., Panagiotakos, G., Barberi, T., Tabar, V., and Studer, L. (2007). Isolation and directed differentiation of neural crest stem cells derived from human embryonic stem cells. Nat. Biotechnol. *25*, 1468–1475.

Love, M.I., Huber, W., and Anders, S. (2014). Moderated estimation of fold change and dispersion for RNA-seq data with DESeq2. Genome Biol. *15*, 550.

Magini, P., Della Monica, M., Uzielli, M.L., Mongelli, P., Scarselli, G., Gambineri, E., Scarano, G., and Seri, M. (2012). Two novel patients with Bohring-Opitz syndrome caused by de novo ASXL1 mutations. Am. J. Med. Genet. A *158A*, 917–921.

Mayor, R., and Theveneau, E. (2013). The neural crest. Development *140*, 2247–2251.

Merzdorf, C.S. (2007). Emerging roles for zic genes in early development. Dev. Dyn. *236*, 922–940.

Noden, D.M., and Trainor, P.A. (2005). Relations and interactions between cranial mesoderm and neural crest populations. J. Anat. *207*, 575–601.

Rada-Iglesias, A., Bajpai, R., Prescott, S., Brugmann, S.A., Swigut, T., and Wysocka, J. (2012). Epigenomic annotation of enhancers predicts transcriptional regulators of human neural crest. Cell Stem Cell *11*, 633–648.

Ramos, T.L., Sanchez-Abarca, L.I., Muntion, S., Preciado, S., Puig, N., Lopez-Ruano, G., Hernandez-Hernandez, A., Redondo, A., Ortega, R., Rodriguez, C., et al. (2016). MSC surface markers (CD44, CD73, and CD90) can identify human MSC-derived extracellular vesicles by conventional flow cytometry. Cell Commun. Signal. *14*, 2.

Rehimi, R., Nikolic, M., Cruz-Molina, S., Tebartz, C., Frommolt, P., Mahabir, E., Clement-Ziza, M., and Rada-Iglesias, A. (2016). Epigenomics-based identification of major cell identity regulators within heterogeneous cell populations. Cell Rep. *17*, 3062–3076.

Simoes-Costa, M., and Bronner, M.E. (2015). Establishing neural crest identity: a gene regulatory recipe. Development *142*, 242–257.

Snider, T.N., and Mishina, Y. (2014). Cranial neural crest cell contribution to craniofacial formation, pathology, and future directions in tissue engineering. Birth Defects Res. C Embryo Today *102*, 324–332.

Studier, F.W. (2005). Protein production by auto-induction in high density shaking cultures. Protein Expr. Purif. *41*, 207–234.

Stem Cell Reports, Volume *12*

Supplemental Information

Pathological ASXL1 Mutations and Protein Variants Impair Neural Crest Development

Friederike Matheus, Ejona Rusha, Rizwan Rehimi, Lena Molitor, Anna Pertek, Miha Modic, Regina Feederle, Andrew Flatley, Elisabeth Kremmer, Arie Geerlof, Valentyna Rishko, Alvaro Rada-Iglesias, and Micha Drukker

Supplemental Table 1. Human induced pluripotent and embryonic stem cell (hiPSC and hESC) lines used in this study. Related to Figures 1-4 and to Supplemental Experimental Procedures. BOS, Bohring-Opitz syndrome; aa, amino acids

**Name**	**Line**	**Generation**<br>(see **Supplemental Experimental Procedures** for details)	**Genomic *ASXL1* background**	**ASXL1 variants expressed**
BOS-iPSC<br>#1-0<br>#1-1<br>#2-0<br>#2-1	iPSC<br>(#1=f,<br>#2=m)	Reprogramming of BOS patient fibroblasts via modified mRNA(-0) and 4-in-1-mini-intronic plasmids(-1)	heterozygous mutation in *ASXL1*<br>(#1: c.2407_2411del5, #2: c.2893C>T)	wildtype ASXL1 (1541 aa) and putative truncated forms (818 aa in #1, 964 aa in #2)
control iPSC	iPSC	reprogramming of control fibroblasts via mmRNA and 4-in-1-mini-intronic plasmids	wildtype: $ASXL1^{+/+}$	wildtype ASXL1
$ASXL1^{+/+}$<br>hESC<br>(control)	iCas9<br>hESC<br>(HUES9)	HUES9 with integrated, tetracycline-inducible Cas9	wildtype: $ASXL1^{+/+}$	wildtype ASXL1
$ASXL1^{PSC/+}$	iCas9<br>hESC	CRISPR/Cas9 mediated deletion of 476 bp in *ASXL1*, leading to premature STOP codon (PSC)	heterozygous mutation in *ASXL1:*<br>c.2419_2894del476	putative truncated ASXL1 (809 aa) and wildtype ASXL1 (1542 aa)
$ASXL1^{PSC/PSC}$	iCas9<br>hESC	CRISPR/Cas9 mediated deletion/ inversion of 475-476 bp in *ASXL1*, leading to premature STOP codon (PSC)	clones A, B, C (homozygous):<br>c.2419_2894del476<br>clone D (compound heterozygous):<br>c.2419_2893inv//<br>c.2419_2893del475	putative truncated ASXL1 (809 aa and/or 824 aa)
GFP-$ASXL1^{+/+}$<br><br>*GFP-*$ASXL1^{PSC/PSC}$	iCas9<br>hESC	stable integration of a constitutively expressed GFP cassette in $ASXL1^{+/+}$ and $ASXL1^{PSC/PSC}$ line	$ASXL1^{+/+}$ (Control) or c.2419_2894del476 ($ASXL1^{PSC/PSC}$)	wildtype ASXL1/putative truncated ASXL1 (809 aa and/or 824 aa)
PB-*ZIC1-*$ASXL1^{+/+}$<br><br>PB-*ZIC1-*$ASXL1^{PSC/PSC}$	iCas9<br>hESC	Stable integration of a tetracycline-inducible Piggybac construct PB-*ZIC1*, bearing *GFP* and the *ZIC1* transcript, in $ASXL1^{PSC/PSC}$ and $ASXL1^{+/+}$ line	$ASXL1^{+/+}$ (Control) or c.2419_2894del476 ($ASXL1^{PSC/PSC}$)	wildtype ASXL1/putative truncated ASXL1 (809 aa and/or 824 aa)
PB-$ASXL1^{PSC}$	iCas9<br>hESC	Stable integration of a tetracycline-inducible Piggybac construct PB-$ASXL1^{PSC}$, bearing *GFP* and the truncated *ASXL1* transcript (N-terminal 2892 bp) in $ASXL1^{+/+}$ hESC	$ASXL1^{+/+}$; random integration of PB-$ASXL1^{PSC}$	Overexpression of truncated ASXL1 construct<br>(964 aa) and endogenous expression of wildtype ASXL1
PB-*ASXL1*<br>in $ASXL1^{+/+}$<br>*and*<br>$ASXL1^{PSC/PSC}$	iCas9<br>hESC	Stable integration of a tetracycline-inducible Piggybac construct PB-*ASXL1*, bearing *GFP* and the wildtype *ASXL1* transcript (4656 bp) in $ASXL1^{+/+}$ and $ASXL1^{PSC/PSC}$ hESC	$ASXL1^{+/+}$ (Control) or c.2419_2894del476 ($ASXL1^{PSC/PSC}$); random integration of PB-*ASXL1*	Overexpression of full-length ASXL1 construct (1541 aa) and endogenous expression of wildtype or putative truncated ASXL1 (809 aa)

Supplemental Table 3. Primers and gRNAs used in this study. Related to Supplemental Experimental Procedures.

Name	Application	Forward sequence	Reverse sequence	Chromosome location
ASXL1 gRNA 1	CRISPR-Cas9: 500 bp deletion	CCATTGTCTGCAGGAACGGT		chr20:32435128
ASXL1 gRNA 2		AGTGAAGTAAGGCTGTCAAG		chr20:32435569
ASXL1-GT	Genotyping PCR	GAGCACCCCTGGAAAGTGTA	TGCTTCAGAGTCTCCGTTGA	chr20:32434755+ 32435685
ASXL1-qPCR	SybrGreen qPCR	GCCACAGGTCAAATGAAGC	GGTCCGAGAGTTGATCAGG	
ASXL1-RT	RT-PCR	TCGCAGACATTAAAGCCCGT	CAGAGGCTGTATCCGTGGA	
ASXL1-seq-F	Sequencing of RT products	GAGCACCCCTGGAAAGTGTA	-	
ASXL1-wildtype allele	SybrGreen qPCR	GAAAGTGATGATGAGGAGCAAGG	ACAGGATCCTTCATAGTGGGA	
ASXL1-mutant allele	SybrGreen qPCR	GGGAAAGTGATGATGAGGAGAC	ACAGGATCCTTCATAGTGGGA	
GAPDH	SybrGreen qPCR	TGCACCACCAACTGCTTAGC	GGCATGGACTGTGGTCATGAG	
OCT4	SybrGreen qPCR	CAATTTGCCAAGCTCCTGAAG	AAAGCGGCAGATGGTCGTT	
SOX2	SybrGreen qPCR	CCTCCGGGACATGATCAGCATGTA	GCAGTGTGCCGTTAATGGCCGTG	
NANOG	SybrGreen qPCR	CCTTCCTCCATGGATCTGCTT	CTTGACCGGGACCTTGTCTTC	
SOX9	SybrGreen qPCR	AGGAAGCTCGCGGACCAGTAC	GGTGGTCCTTCTTGTGCTGCAC	
SOX10	SybrGreen qPCR	ATGAACGCCTTCATGGTGTGGG	CGCTTGTCACTTTCGTTCAGCAG	
PAX3	SybrGreen qPCR	GGCTTTCAACCATCTCATTCCCG	GTTGAGGTCTGTGAACGGTGCT	
SLUG	SybrGreen qPCR	ATCTGCGGCAAGGCGTTTTCCA	GAGCCCTCAGATTTGACCTGTC	
TFAP2A	SybrGreen qPCR	GACCTCTCGATCCACTCCTTAC	GAGACGGCATTGCTGTTGGACT	
GBX2	SybrGreen qPCR	GCGGAGGACGGCAAAGGCTTC	GTCGTCTTCCACCTTTGACTCG	
E-CAD	SybrGreen qPCR	GCCTCCTGAAAAGAGAGTGGAAG	TGGCAGTGTCTCTCCAAATCCG	
N-CAD	SybrGreen qPCR	CCCACACCCTGGAGACATTG	GCCGCTTTAAGGCCCTCA	
ZIC1	SybrGreen qPCR	GATGTGCGACAAGTCCTACACG	TGGAGGATTCGTAGCCAGAGCT	
NR2F1	SybrGreen qPCR	TGCCTCAAAGCCATCGTGCTGT	CAGCAGCAGTTTGCCAAAACGG	
NR2F2	SybrGreen qPCR	TGCACGTTGACTCAGCCGAGTA	AAGCACACTGAGACTTTTCCTGC	

Supplemental Table 4. Antibodies used in this study. Related to Supplemental Experimental Procedures.

Name	Application	Manufacturer	Cat.#
ASXL1 (clone 4F6)	Western Blot	This study	-
ACTIN	Western Blot	Cell Signaling	3700
Oct4	Immunocytochemistry	Cell Signaling	2840 (C30A3)
Sox2	Immunocytochemistry	Cell signaling	2748
Nanog	Immunocytochemistry	Abcam	ab21603
Lin28a	Immunocytochemistry	Cell Signaling	3978 (a177)
TFAP2A	Immunocytochemistry	Santa Cruz	sc-184X
Sox9	Immunocytochemistry	supplied by M. Götz	-
Ki67	Immunocytochemistry	supplied by M. Götz	-
p75NTR	Immunocytochemistry	R&D	MAB367
ZIC1	Western Blot	R&D	AF4978-SP
H3	Western Blot	Abcam	ab1791
CD73-APC (clone AD2)			
CD90-FITC (clone DG3)			
CD105-PE (clone 43A4E1)			
CD45-PerCP (clone 5B1)			
IgG1-FITC (clone: IS5-21F5)	Flow Cytometry	Miltenyj Biotec (human MSC Phenotyping Kit)	130-095-198
mouse IgG1-PE (clone: IS5-21F5)			
mouse IgG1-APC (clone: IS5-21F5)			
IgG1-PerCP (clone: IS5-21F5)			
IgG2a-PerCP (clone S43.10)			

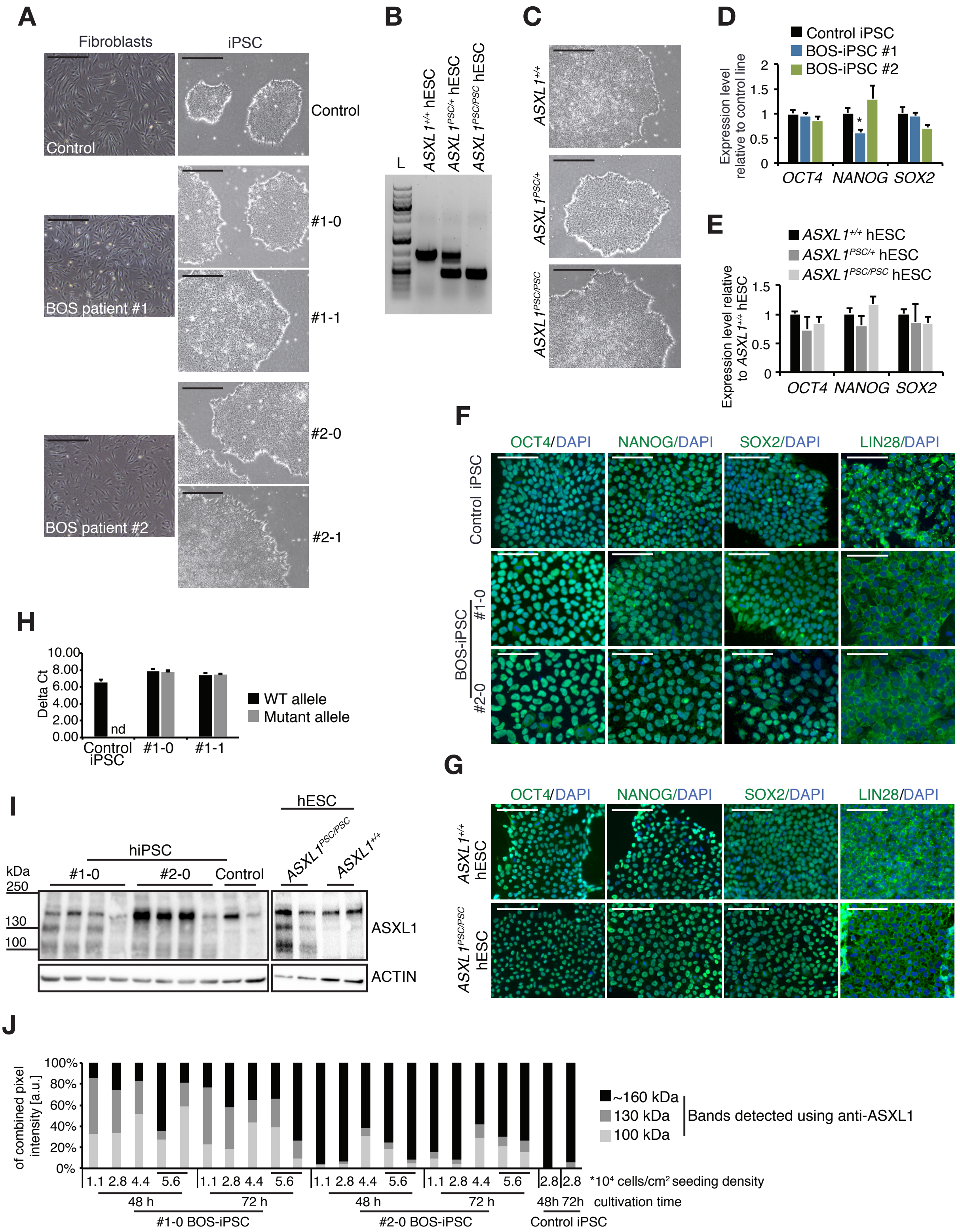

Supplemental Figure S1. Generation of human pluripotent stem cell models for BOS. Related to Figure 1.

Supplemental Figure S1. Generation of human pluripotent stem cell models for BOS. Related to Figure 1. (A) Bright-field images of control and BOS-patient-derived iPSC lines and parental fibroblasts. (B) Analysis of the *ASXL1* locus showing homozygous or heterozygous 476 bp deletion in $ASXL1^{PSC/PSC}$ hESC, clone A, and the $ASXL1^{PSC/+}$ hESC clone, respectively; L, molecular weight marker. (C) Bright-field images of $ASXL1^{+/+}$ hESC, $ASXL1^{PSC/+}$ and $ASXL1^{PSC/PSC}$ modified hESC clones. Overt morphological differences of *ASXL1* mutant to $ASXL1^{+/+}$ type were not observed. (D-G) *ASXL1* mutations do not generally affect pluripotency. This was shown by qPCR analysis (D, E) and immunocytochemical analysis (F, G) of core pluripotency transcription factors in undifferentiated BOS-iPSC and control iPSC lines (D, F) and in $ASXL1^{PSC}$ hESC lines relative to the $ASXL1^{+/+}$ hESC line (E, G). In (D, E), pairwise comparisons between mutant and control lines did not yield statistically significant differences, except for *NANOG* as indicated ($n \geq 3$, different clones/passages). (H) Analysis of mutant and wildtype alleles using primers spanning the 5 bp deletion in BOS iPSC #1 (n=3, different passages, n.d. not detected) confirmed comparable expression levels for both transcripts in BOS-iPSC #1 lines. Samples derived from control iPSCs served as negative control for the detection of the mutant transcript; Ct values were normalized to *GAPDH* to obtain delta Ct values. (I) Extended image of the Blot shown in Fig. 1F, demonstrating detection of several putative protein forms by the monoclonal anti-ASXL1 antibody, which are expressed at varying levels in BOS-iPSC lines and the $ASXL1^{PSC/PSC}$ line. (J) Quantification of bands shows variable levels of different protein variants detected using the monoclonal antibody. Analysis of two different Western Blot experiments, one of which is shown in (I), of BOS- and control iPSC lines seeded at different densities and harvested 48 h or 72 h later. For (A) and (C), scale bar indicates 500 μM. In (F) and (G), scale bars represent 200 μM.

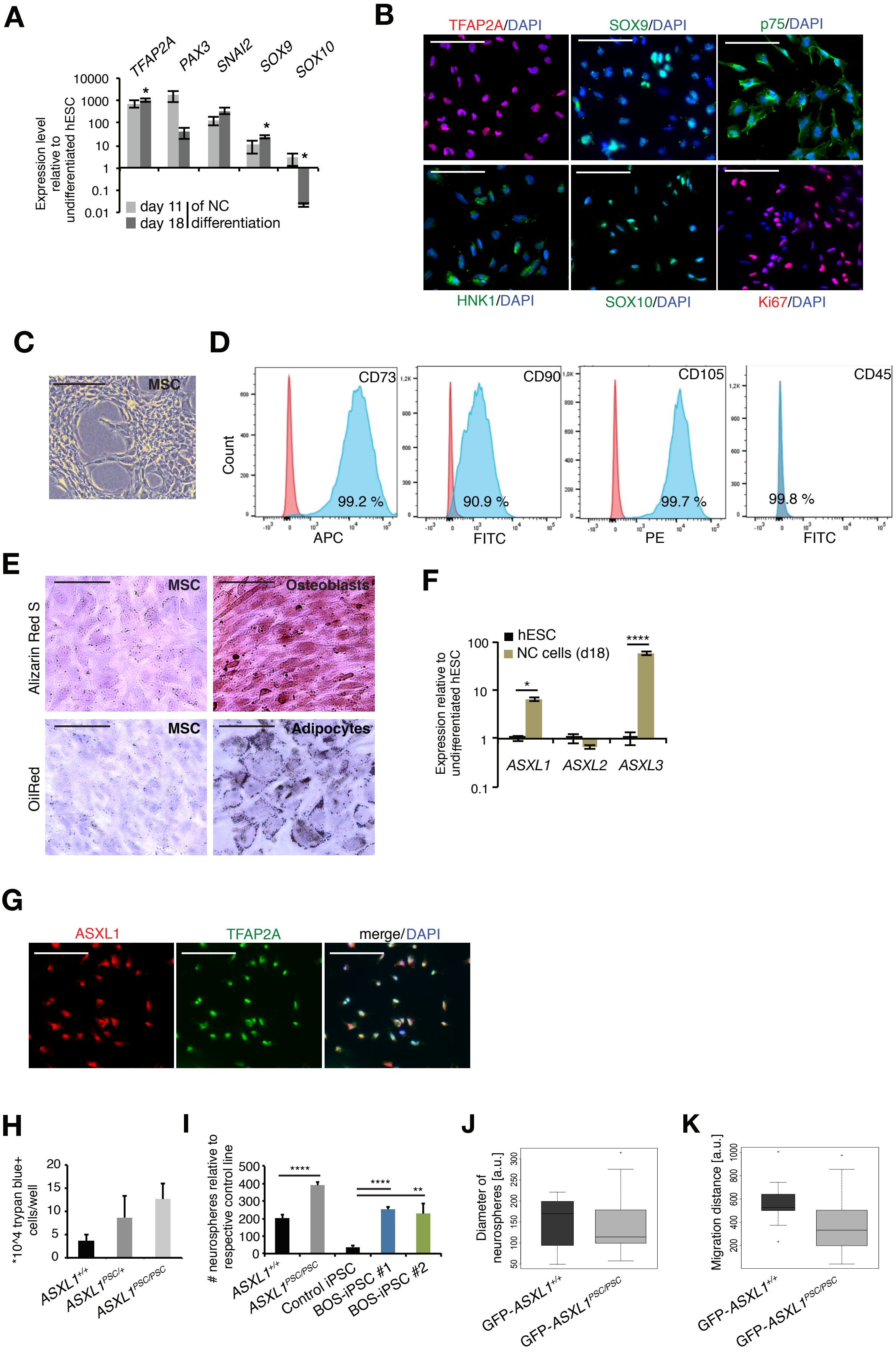

Supplemental Figure S2. Evidence for differentiation of neural crest cells and analysis of the delamination defect in ASXL1 mutant cells. Related to Figure 2.

Supplemental Figure S2. Evidence for differentiation of neural crest cells and analysis of the delamination defect in *ASXL1* mutant cells. Related to Figure 2. (A,B) Analysis of neural crest (NC) markers in *ASXL1*$^{+/+}$ hESC derived NC cultures by qPCR (A) and immunocytochemistry (B). NC markers including *TFAP2A* and *SNAI2* were highly induced, however pairwise comparison of expression levels in undifferentiated cells and NC cells did not yield significant differences, except for *TFAP2A*, *SOX9* and *SOX10* at day 18 as indicated, due to moderate variation of low transcript levels in undifferentiated cells. Early primitive streak markers *SOX17*, *FOXA2*, *T* (*Brachyury*) and *PAX6* were not detected (not shown; *n*=3 independent experiments). (B) Representative immunocytochemical stainings of NC markers and the proliferation marker Ki67 in passaged NC-like cells (day 18 of differentiation, *n*=3 independent experiments). (C-E) Cells differentiated from NC-like cells by a protocol developed to induce differentiation into mesenchymal stem cells (MSCs) exhibited mesenchymal morphology in bright field microscopy (C), uniformly expressed a cohort of MSC-surface markers but not the hematopoietic marker CD45 as determined by flow cytometry when gates were set by the same IgG control (D), and when exposed to respective differentiation cues, terminally differentiated to osteoblasts and adipocytes as assayed by Alizarin Red and Oil Red O staining, respectively (E; *n*=4 independent experiments). (F) Upregulation of *ASXL1* and *ASXL3* in control hESC-derived NC cells (day 18) relative to undifferentiated hESCs, as demonstrated by qPCR analysis of the *ASXL* genes (n=3 different passages in separate experiments). (G) Representative immunocytochemical staining showing co-localization of ASXL1 and the NC marker TFAP2A in passaged *ASXL1*$^{+/+}$ hESC-derived NC-like cells (day 18 of differentiation, *n*=3 independent experiments). (H) Quantification of trypan blue positive cells in supernatants of day 7 NC cultures derived from *ASXL1*$^{+/+}$, *ASXL1*$^{PSC/+}$ and *ASXL1*$^{PSC/PSC}$ hESC lines (*n*=3 different clones/passages). (I) Quantification of neurospheres (total numbers including attached and floating neurospheres) derived from ASXL1$^{PSC/PSC}$ (clones A-D) relative to *ASXL1*$^{+/+}$ hESCs, and from BOS-iPSC lines relative to control iPSCs, after 7 days of NC differentiation (*n*≥3 different clones/passages in separate experiments). (J, K) Analysis of neurospheres based on transplantation experiments in Fig. 2D; *ASXL1*$^{+/+}$: *n*=9, *ASXL1*$^{PSC/PSC}$: *n*=21 embryos. (J) Diameter of neurospheres derived from *GFP-ASXL1*$^{PSC/PSC}$ and *GFP- ASXL1*$^{+/+}$ hESC clones, 48 h following transplantation into chicken embryos; *p*=0.45. (K) Distance between neurospheres and furthest migrated cell, 48 h after transplantation; *p*=0.05. For (B, C, E and G): scale bars indicate 100 μM.

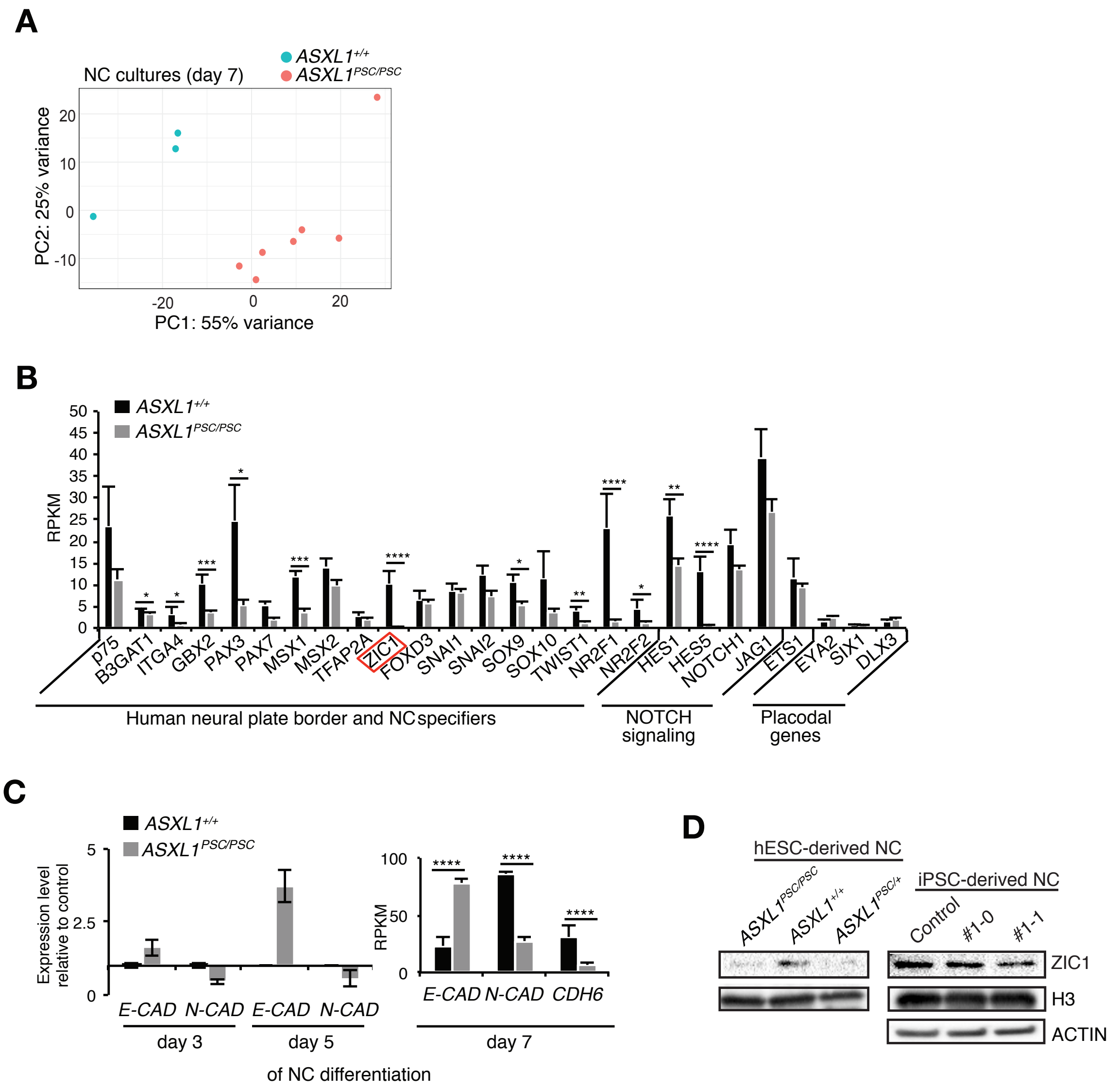

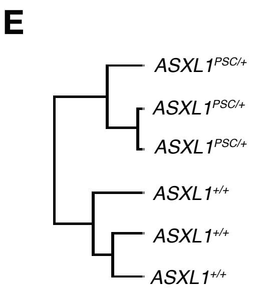

F

Gene	fold change	p(adj)
FOXG1	29,52	0,01920
LHX1	28,50	0,04661
ZIC1	27,89	0,02147
MIXL1	16,53	0,00512
AMN	14,96	0,02282
HYDIN	11,56	0,00191
AC104653.1	9,95	0,02702
TEKT2	8,21	0,00512
MT-TS2	7,98	0,00214
LINC00106	7,55	0,01717

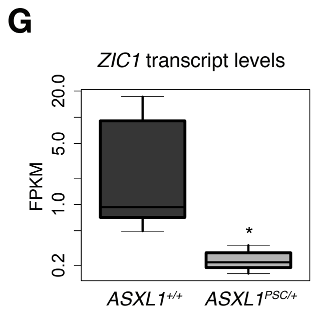

Supplemental Figure S3. Misregulation of NC regulatory networks in *ASXL1*^{PSC} NC progenitors. Related to Fig. 3.

Supplemental Figure S3. Misregulation of NC regulatory networks in *ASXL1*^{PSC} NC progenitors. Related to Fig. 3. (A) Principal component analysis of the global transcriptomes in samples obtained from *ASXL1*$^{PSC/PSC}$ and *ASXL1*$^{+/+}$ hESC line-derived day 7 NC progenitors (*ASXL1*$^{+/+}$: *n*=3; *ASXL1*$^{PSC/PSC}$ clone A and C: *n*=2, *ASXL1*$^{PSC/PSC}$ clone D: *n*=3; based on 3 independent differentiation experiments). (B) Confirmation of NC identity in day 7 progenitor cultures based on expression of published human NC markers and NOTCH signaling pathway members, and low transcript levels of placode-associated genes [based on experiment in (A)]. *ETS1* is a cranial NC cell transcription factor. *ASXL1*$^{PSC/PSC}$-derived cultures showed reduced expression levels of several NC specifiers and NOTCH factors, *ZIC1* was the most significantly downregulated gene. These data highlight the misregulation of NC pathways in *ASXL1*$^{PSC/PSC}$ clones. RPKM, reads per kilobase per million mapped reads. (C) Quantification *of E-Cadherin* (*E-CAD*) and *N-Cadherin* (*N-CAD*) expression at day 3 and 5 (qPCR, *n*=2 different clones/passages), and of *E-CAD, N-CAD* and *Cadherin-6 (CDH6)* expression at day 7 [RNA-seq, based on experiment in (A)] of NC differentiation from *ASXL1*$^{PSC/PSC}$ compared to *ASXL1*$^{+/+}$ cells. Misregulation of *E-CAD* and *N-CAD* in *ASXL1*$^{PSC/PSC}$ cultures suggests perturbation of the Epithelial-to-Mesenchymal Transition (EMT) required for NC delamination. (D). Representative Western Blots for the detection of ZIC1 protein in hESC clones and iPSC-derived NC progenitors at day 7 of differentiation (related to Fig. 3F). H3 controls in hESC-derived samples are the same as for Fig. 4A, as detection of ZIC1 was performed on the same Blots. (E-G) Analysis of global transcriptomes in NC progenitors derived from *ASXL1*$^{PSC/+}$ and *ASXL1*$^{+/+}$ hESC lines confirms general transcriptional deregulation and impaired activation of *ZIC1* in the heterozygous *ASXL1* mutant line. Based on 3'RNA sequencing of samples derived from day 7 NC cultures (*n*=3 different passages for each line, based on samples shown in Fig. 3D). (E) Hierarchical clustering of mapped sequencing reads from *ASXL1*$^{PSC/+}$ and *ASXL1*$^{+/+}$ NC progenitors. (F) The list of topmost downregulated genes in *ASXL1*$^{PSC/+}$ compared to *ASXL1*$^{+/+}$ NC cultures includes *ZIC1* as one of the most strongly reduced transcripts, supporting results obtained in *ASXL1*$^{PSC/PSC}$ NC progenitors. (G) *ZIC1* transcript levels in *ASXL1*$^{PSC/+}$ and *ASXL1*$^{+/+}$ NC progenitors. FPKM, fragments per kilobase per million mapped reads.

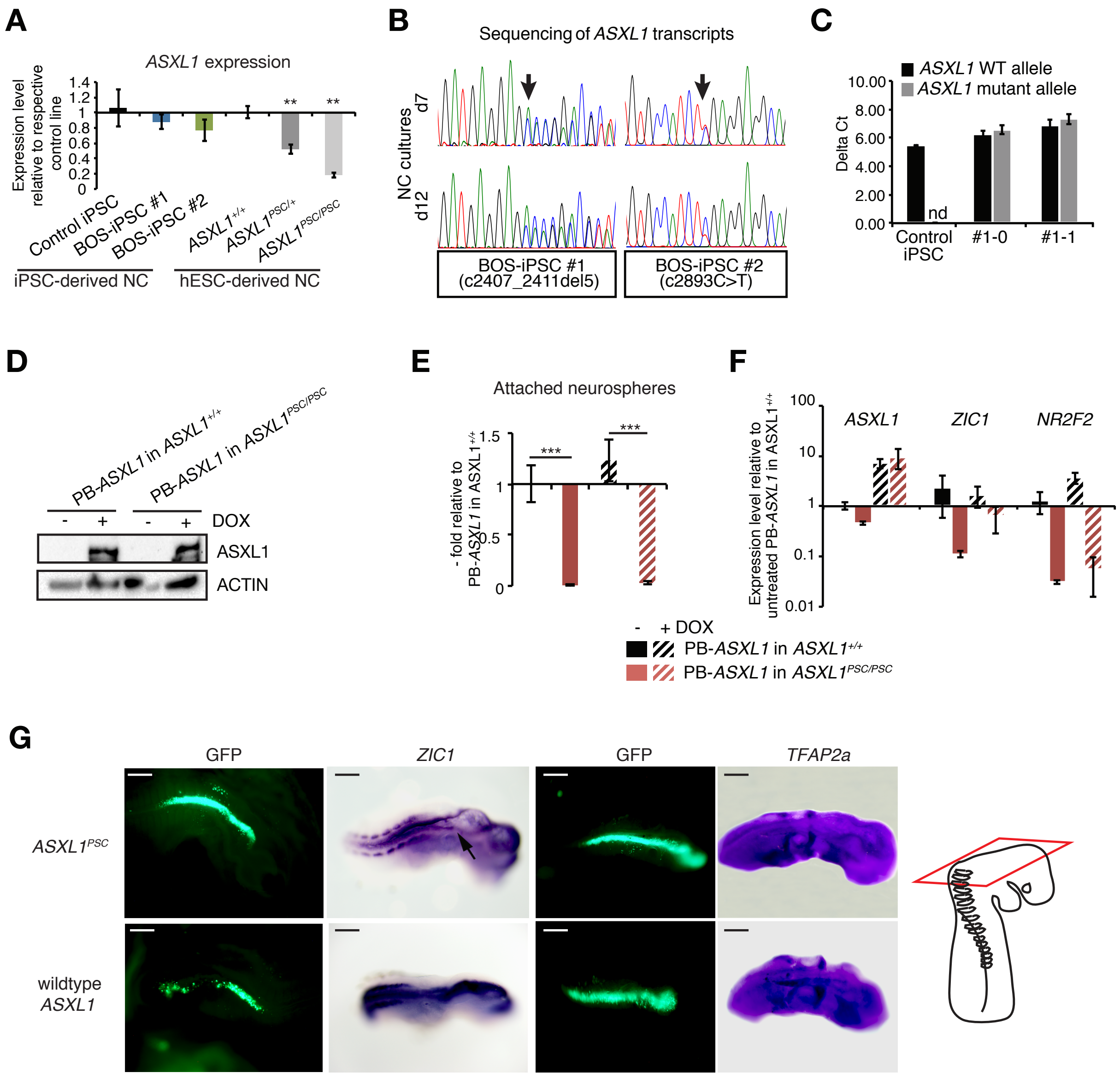

Supplemental Figure S4. Investigation of molecular mechanisms underlying NC development defects in BOS models. Related to Fig. 4.

Supplemental Figure S4. Investigation of molecular mechanisms underlying NC development defects in BOS models. Related to Fig. 4. (A) qPCR analysis of *ASXL1* transcript in day 7 NC cultures derived from BOS-iPSC, $ASXL1^{PSC/+}$ and $ASXL1^{PSC/PSC}$ hESC lines relative to the respective control lines. Primers were specific to Exon 4, thus detecting wildtype and mutant transcripts (*n*=3-6 different clones/passages). (B) Sequences of reverse transcribed *ASXL1* transcripts, isolated from day 7 and day 12 NC cultures of BOS-iPSC #1-0 (day 12), #1-1 (day 7), #2-0 (day 12) and #2-1 (day 7), confirm expression of the mutant transcripts in NC progenitors derived from both patient lines. (C) Discrimination of levels of mutant and wildtype *ASXL1* alleles in samples based on (A), via qPCR, using primers spanning the 5 bp deletion in BOS iPSC #1 (*n*=3 different passages, n.d. not detected). Ct values were normalized against *GAPDH* to obtain delta Ct values. (D-F) Ectopic expression of wildtype *ASXL1* does not rescue the NC differentiation defect in $ASXL1^{PSC/PSC}$ hESC. (D) Western Blot analysis showing the ectopic expression of ASXL1 in DOX-treated $ASXL1^{+/+}$ and $ASXL1^{PSC/PSC}$ cells, harboring stably integrated PB-*ASXL1* expression constructs. (E) Quantification of attached neurospheres with emigrating cells in lines from (D) at day 7 of NC differentiation, left untreated or treated by DOX to ectopically express *ASXL1* from day 1 to day 7 (*n*=10 from 3 independent experiments). Ectopic *ASXL1* expression did not lead to significant improvement of neurosphere attachment in $ASXL1^{PSC/PSC}$ lines. (F) Quantification of *ASXL1, ZIC1* and *NR2F2* expression in lines from (D), with or without DOX treatment from day 1 to day 7 of differentiation. While *ASXL1* transcript levels are comparable in the $ASXL1^{+/+}$ and $ASXL1^{PSC/PSC}$ clones after induction of *ASXL1* expression by DOX treatment, *ZIC1* levels were not entirely rescued, and *NR2F2* levels remained unchanged. *n*=3 different passages; pairwise comparisons between treatments or between genotypes did not yield statistically significant differences due to moderate variations between replicates. (G) Detection of TFAP2A and ZIC1 by *in situ* hybridization in chicken embryos electroporated with plasmids encoding for truncated ($ASXL1^{PSC}$) or wildtype chicken ASXL1 coupled to GFP; related to Fig. 4D. Whole mount images show HH15 embryos as demonstrated by the schematic illustration. The arrow indicates reduced *ZIC1* expression in embryos expressing truncated ASXL1. *n*=2 embryos. Scale bar represents 200 μM.

Supplemental Experimental Procedures

Generation of monoclonal antibodies.

Cloning of construct, protein expression and purification. A 5'fragment of the coding sequence of *ASXL1* (bp 1-1854) was amplified by PCR from human cDNA and cloned into pETM13/LIC containing a C-teminal His_6-tag (supplied by A. Geerlof). The pETM-13/ASXL1 construct was transformed into *E. coli* strain BL21 (DE3) and cultured at 20°C in ZYM 5052 auto-induction medium (Studier, 2005). Cells were harvested after reaching saturation, lysed by sonication, and lysates clarified by centrifugation and filtration (0.2 μM) and applied to a 5 ml HiTrap Chelating HP column (GE Healthcare) using a Äkta Purifier (GE Healthcare). Bound proteins were eluted, fractions containing protein pooled and subsequently applied to size exclusion chromatography using a HiLoad 16/600 Superdex 200 column (GE Healthcare). The fractions containing ASXL1 were pooled and stored at 4°C.

Antibody production. To generate monoclonal antibodies against ASXL1, Lou/c rats were immunized with purified his-tagged human ASXL1 protein (aa 1-618) using standard procedures as described (Feederle et al., 2016). Hybridoma supernatants were validated in enzyme-linked immunoassay (capture and detection ELISA) and by Western blot analysis on control hESC and PB-$ASXL1^{PSC}$ hESC overexpressing truncated ASXL1 protein. The hybridoma cells of ASXL1-reactive supernatants were cloned three times by limiting dilution. Experiments in this study were performed with hybridoma culture supernatant of ASXL1 clones 12F9 and 4F6 (both rat IgG2a/k).

Pluripotent stem cell lines

All iPSC and HUES9 hESC lines were maintained as feeder-free cultures in mTeSR1 medium (STEMCELL Technologies) or StemMACS iPS-Brew XF (Miltenyi Biotec) on Matrigel (Corning)– or Geltrex (Life Technologies)- coated cell culture plates as described (Ludwig et al., 2006) .

Generation of iPSC lines.

mRNA-mediated reprogramming. Bohring-Opitz Syndrome (BOS) fibroblasts were derived from skin biopsies of BOS patients, age 5 and 7 years at the day of procurement [informed consent of the patient's parents given in the original study (Magini et al., 2012)]. Control fibroblasts (ATCC CRL-2522) and BOS fibroblasts were seeded onto NuFF3-RQ cells (GlobalStem, GSC-3404) at day 1; medium was Pluriton Reprogramming Medium (Stemgent) supplemented with 500ng/ml carrier-free B18R Recombinant Protein. From day 3-18, a modified

mRNA cocktail containing *OCT4, SOX2, KLF4, LIN28*, and *C-MYC* mmRNAs at a 3:1:1:1:1 stoichiometric ratio was transfected daily for 4 hours using the Lipofectamine RNAiMAX Transfection Reagent (Thermo Fisher Scientific), followed by washes and addition of fresh reprogramming medium supplemented with B18R. The mmRNA factors were modified with 5-Methyl CTP, Pseudo-UTP, ARCA cap and a 150 nt poly-A tail (provided by the RNA CORE of the Houston Methodist Hospital). Medium was changed to STEMPRO hESC SFM (Thermo Fisher Scientific) from day 16 to day 21, when iPSC colonies were harvested, plated on γ-irradiated mouse embryonic fibroblasts and grown in STEMPRO for 10 additional passages before adapting the iPSCs to a feeder-free culture system.

*Episomal-based reprogramming***.** Control fibroblasts (courtesy of Prof. Magdalena Goetz) and BOS fibroblasts were transfected with 6 or 12 μg of plasmid DNA (MIP 247 CoMiP 4in1 with shRNA p53: pCXLE-hMLN; Addgene #63726 and #27079) using the Nucleofector 2b Device (Lonza) with the MEF 1 Nucleofector Kit (Lonza) and T-020 program. Transfected cells were plated on Matrigel-coated plates and incubated overnight in fibroblast medium supplemented with 10% HyClone Fetal Bovine Serum, 0.2 mM sodium butyrate and 50 µg/mL ascorbic acid. On day 2, medium was changed to Essential 7 medium supplemented with 0.2 mM sodium butyrate and 50 µg/mL ascorbic acid. Between days 15-20, culture conditions were switched to Essential 8 medium and around days 21–30, iPSC colonies were manually selected under the microscope.

Generation of *ASXL1*^{PSC} hESC lines

1x10E6 iCas9 HUES9 hESC (Gonzalez et al., 2014) were electroporated with 3 μg of two gRNA expression plasmids (Supplemental Table 3) using the P3 Primary Cell 4D Nucleofector X Kit (Lonza). Cas9 expression was induced after nucleofection by addition of 1 μg/ml doxycycline for 48h and single cells were plated on Matrigel-coated 15 cm plates in mTeSR1 medium containing 10 μM ROCK-inhibitor (Y-27632; R&D Systems). After 7-10 days, colonies were isolated, passaged, tested for targeted deletion via PCR (for primers see Supplemental Table 3) and Sanger sequencing, and maintained as individual lines.

Generation of hESC lines with stable integration of expression vectors

Generation of lines harboring inducible PB-*ASXL1*^{PSC}, PB-*ASXL1 and* PB-*ZIC1 expression plasmids*

1x10E6 iCas9 HUES9 (Gonzalez et al., 2014) and *ASXL1*$^{PSC/PSC}$ hESC were electroporated as described with a PiggyBac vector containing either the truncated *ASXL1* cDNA sequence (N-terminal 2892 bp), the full-length human ASXL1 cDNA (4656 bp; PB-*ASXL1*), or the *ZIC1* cDNA sequence (amplified from *ZIC1* Human cDNA clone, Biocat), followed by P2A-GFP. The plasmids contained a tetracycline-inducible promoter system and a

Hygromycin resistance gene. A plasmid expressing the Piggybac transposase was co-transfected. Stably integrated clones were selected by 2 weeks' treatment with 50 μg/ml Hygromycin B. To induce overexpression, cells were treated with doxycycline for 24h-48h. Expression of truncated or wildtype ASXL1, or of ZIC1, was tested by qPCR, Western blotting and immunocytochemistry.

Generation of GFP-ASXL1$^{PSC/PSC}$ and GFP-ASXL1$^{+/+}$ hESC lines

ASXL1$^{PSC/PSC}$ and *ASXL1$^{+/+}$* hESC lines were transfected as described above with a transposase-expressing vector together with a PiggyBac vector harboring a *GFP* expression cassette regulated by the CAG promoter. Homogenous cultures of GFP-expressing cells were obtained by sorting using a FACS AriaIII (BD Biosciences) with non-transfected cells serving as a negative control.

Neural crest differentiation and analysis.

NC differentiation. Differentiation to NC was performed essentially as described (Bajpai et al., 2010). Briefly, pluripotent stem cell lines were detached and placed into low attachment plates in NC induction medium to form aggregates (neurospheres). At day 4, neurospheres were plated onto uncoated tissue culture dishes, until NC-like cells had emigrated and could be passaged to dishes coated with 5 μg/ml fibronectin after removal of the neurospheres (around day 11). From then, cells were cultured in maintenance medium, to which 50 pg/ml BMP2 and 3 μM CHIR99021 were added after passage 2. The number of attached, outgrowing neurospheres was quantified at day 7 and related to the total number of floating and attached neurospheres. Attached neurospheres that exhibited uniform emigration of cell sheets or at least 10 NC cells or cell clusters in their direct vicinity (approximately 100 µm apart) were classified as neurospheres with delaminating, migrating NC cells.

MSC differentiation. For conversion to MSCs, NC cells (>passage 2) were cultivated on uncoated dishes in human StemMACS MSC Expansion Media (Miltenyi Biotec) for around 10 days before morphology was assessed by brightfield microscopy. To test for MSC marker expression, NC-derived MSC were dissociated with 0.25% Trypsin-EDTA and stained for positive and negative MSC markers using the human MSC Phenotyping Kit (Miltenyj Biotec). For controls, cells were incubated with the kit's isotype control cocktail. The analysis was performed on a FACS AriaIII.

Terminal differentiation to osteoblasts/adipocytes. NC-derived MSC were cultivated to 90 % confluence in 12- or 24-well plates, subsequently, medium was changed to human StemMACS OsteoDiff Media or human StemMACS AdipoDiff Media (Miltenyi Biotec) to induce osteoblast and adipocyte differentiation, respectively, while control wells were kept in MSC Expansion Media. After 3 weeks, staining of osteoblasts and controls was

performed by 45 min fixation with 10% Formalin, followed by incubation with 20 mg/ml Alizarin Red S and subsequent washes with de-ionized water. Adipocytes and control cells were fixed first with 10 % Formalin for 45 min, then with 60 % 2-propanol for 5 min. Staining was performed by 3 mg/ml Red Oil O solution for 10 min followed by Mayer's Hematoxylin solution for 5 min, with subsequent tap-water washes. Pictures were taken under a brightfield microscope.

Manipulation of chicken embryos

According to German animal care guidelines, no IACUC (Institutional Animal Care and Use Committee) approval was necessary to perform chicken embryo experiments. Fertilized chicken eggs (*Gallus gallus domesticus*) were obtained from a local breeder (LSL Rhein-Main) and incubated at 37°C and 80% humidity in a normal poultry egg incubator. Following microsurgical procedures, the eggs were re-incubated until the embryos reached the desired developmental stages according to the staging system of HH (Hamburger and Hamilton, 1992).

In ovo transplantation of neurospheres

Neurospheres obtained at day 5 of NC differentiation were inserted into the developing anterior neural region of chicken embryos (8-10 somite stage, HH10) and operated eggs were sealed with medical tape and re-incubated until stage HH22, when the embryos were isolated and analyzed under a fluorescence stereo microscope (Olympus SZX 16; details outlined in Supplemental Methods). Distance between transplanted neurospheres and furthest migrated cell, diameter of neurospheres and the total number of migrated cells were determined using the Fiji software (Schindelin et al., 2012).

In ovo electroporation of chicken embryos

The truncated chicken (*Gallus gallus*) *ASXL1* cDNA sequence (Gg-*ASXL1*^{PSC}; N-terminal 2445 bp), the truncated human *ASXL1* cDNA sequence (h*ASXL1*^{PSC}; N-terminal 2892 bp) or the full-length chicken *ASXL1* cDNA sequence (Gg-*ASXL1*; 4617 bp) were cloned into a pCIG vector harboring a *GFP* coding sequence coupled to the *T2A* cleavage signal. The plasmids were mixed with fast green solution (Sigma) at a 2:1 ratio to ease the detection of the injection site and microinjected into the target site of the developing brain and neural tube of HH9-10 chicken embryos. For electroporation, electrodes were placed on each side of the microinjected brain and neural tube, and five square pulses of 35 V within 20 ms width were applied to each embryo using the Intracel TSS20 OVODYNE Electroporator. For control experiments, the vector expressing only GFP or the full-length chicken ASXL1 sequence was electroporated as described above.

Following electroporation, the eggs were sealed with medical tape and re-incubated until the desired developmental stages of chicken embryos (HH19 and HH24-25), to be imaged under a fluorescence stereo microscope.

In situ hybridization in chicken embryos

Chicken embryos were electroporated at stage HH9 with the plasmids described above and fixed at stage HH15. Fixation and subsequent *in situ* hybridization were performed according to the same protocol and using the same *ZIC1* and *TFAP2A* probes as described before (Rehimi et al., 2016).

Gene expression analysis

RT-PCR and qPCR. RNA was extracted using the RNeasy Mini Kit (Qiagen) and reverse transcribed using the SuperScript III kit (Life Technologies). Power SYBR Green PCR Master Mix or Taqman Gene Expression Master Mix (Life Technologies) were used for qPCR on a QuantStudio 12k Flex (Life Technologies). Primers are listed in Supplemental Table 3. Ct values were normalized against the *GAPDH* housekeeping control to obtain delta Ct values, based on which delta delta Ct values and relative expression levels were calculated.

Taq Polymerase (Qiagen) was used in RT-PCR, and products were subjected to Sanger sequencing.

RNA sequencing. RNA from day 7 NC progenitor cultures (1 well of a 6-well plate per replicate; for global RNA-seq: 3 independent differentiation experiments, one clone of *ASXL1*$^{+/+}$ and 3 clones of *ASXL1*$^{PSC/PSC}$, for 3' RNA-seq: 3 different passages of *ASXL1*$^{+/+}$ and *ASXL1*$^{PSC/+}$ clones) was extracted using the RNeasy Mini Kit and quality assessed using an Agilent 2100 Bioanalyzer with the RNA 6000 Pico Kit (Agilent). For global RNA-seq, libraries were prepared using 1 μg of total RNA with the TruSeq Stranded Total RNA LT Library Prep Kit (with Ribo- Zero Human/Mouse/Rat; Illumina) according to the supplied protocol. Libraries were analyzed with the High Sensitivity DNA Kit (Agilent), pooled, diluted 1:10 and subjected to single-end sequencing for 75 cycles on a NextSeq with the NextSeq 500/550 v2 reagent cartridge (Illumina). For 3'RNA-seq, 0.5 μg of RNA was used for library preparation with the QuantSeq 3' mRNA Library Prep Kit FWD for Illumina (Lexogen) according to the manufacturer's specifications. The libraries were sequenced on the Illumina HiSeq2500 platform, producing 51 nt single-end reads.

RNA-seq data analysis and statistics. Quality of the sequencing run was confirmed via QC reports on the Illumina BaseSpace platform and the FastQC tool provided on the Galaxy platform (Afgan et al., 2016). Reads were mapped to the hg19 genome using TopHat (global RNA-seq) or mapped to GRCh38 using STAR (Dobin et al., 2013)(3'RNA-seq). Read count was performed using Cufflinks 2.2.1 (3'RNA-seq data) or, in the case of

global RA-seq, using the featureCounts (v1.5.0) function of the Subread package (Liao et al., 2014) and the hg19 human gene annotation. Differential gene expression analysis was performed with the DESeq2 package (Love et al., 2014) in R v3.3.2; genes with a maximum read count of zero were considered to be non-expressed and thus removed from further analysis. PCA Plots and Volcano Plots were built using the ggplot2, dplyr and ggrepel packages in R, respectively. Hierarchical clustering was performed using the Seqmonk software (v.1.44.0). To characterize misregulated gene sets, the list of significantly downregulated transcripts (padj<0.05) was submitted to the Genomatix Software Suite GeneRanker under *http://www.genomatix.de*.

Immunocytochemistry

Cells were fixed with 4% Formaldehyde for 10-15 min, permeabilized and blocked and subsequently stained with primary antibodies at 4° C overnight. Secondary antibodies were added for 1 hour at room temperature and slides were mounted and analyzed by fluorescence microscopy. Antibodies are listed in Supplemental Table 4.

Western Blotting

Total protein lysates were prepared in RIPA buffer, separated via SDS PAGE and wet-blotted on nitrocellulose membranes, which were blocked for 1 h and treated with primary antibodies overnight at 4°C. Secondary antibodies were added to membranes for 1 h at room temperature, and detection was done on a ChemiDoc XRS using Clarity Western ECL Substrate (Bio-Rad Laboratories). Intensities were normalized to Actin or H3 band intensities to determine relative protein levels. Antibodies are listed in Supplemental Table 4.

Statistical analysis

For pairwise comparison of means, Shapiro-Wilk test was applied to evaluate normal distribution of samples, and if given, Welch's t-test was applied. Otherwise, Wilcoxon Rank-Sum test was used to test for statistical significance. P-values were indicated in figures as follows: ns - $p>0.05$, * $p<0.05$, ** $p<0.01$, *** $p<0.001$, ****$p_{adj}<0.0001$.

Supplemental References

Afgan, E., Baker, D., van den Beek, M., Blankenberg, D., Bouvier, D., Cech, M., Chilton, J., Clements, D., Coraor, N., Eberhard, C., *et al.* (2016). The Galaxy platform for accessible, reproducible and collaborative biomedical analyses: 2016 update. Nucleic acids research *44*, W3-W10.

Bajpai, R., Chen, D.A., Rada-Iglesias, A., Zhang, J., Xiong, Y., Helms, J., Chang, C.P., Zhao, Y., Swigut, T., and Wysocka, J. (2010). CHD7 cooperates with PBAF to control multipotent neural crest formation. Nature *463*, 958-962.

Dobin, A., Davis, C.A., Schlesinger, F., Drenkow, J., Zaleski, C., Jha, S., Batut, P., Chaisson, M., and Gingeras, T.R. (2013). STAR: ultrafast universal RNA-seq aligner. Bioinformatics *29*, 15-21.

Feederle, R., Gerber, J.K., Middleton, A., Northrup, E., Kist, R., Kremmer, E., and Peters, H. (2016). Generation of Pax1/PAX1-Specific Monoclonal Antibodies. Monoclonal antibodies in immunodiagnosis and immunotherapy.

Gonzalez, F., Zhu, Z., Shi, Z.D., Lelli, K., Verma, N., Li, Q.V., and Huangfu, D. (2014). An iCRISPR platform for rapid, multiplexable, and inducible genome editing in human pluripotent stem cells. Cell stem cell *15*, 215-226.

Hamburger, V., and Hamilton, H.L. (1992). A series of normal stages in the development of the chick embryo. 1951. Developmental dynamics : an official publication of the American Association of Anatomists *195*, 231-272.

Liao, Y., Smyth, G.K., and Shi, W. (2014). featureCounts: an efficient general purpose program for assigning sequence reads to genomic features. Bioinformatics *30*, 923-930.

Love, M.I., Huber, W., and Anders, S. (2014). Moderated estimation of fold change and dispersion for RNA-seq data with DESeq2. Genome biology *15*, 550.

Ludwig, T.E., Levenstein, M.E., Jones, J.M., Berggren, W.T., Mitchen, E.R., Frane, J.L., Crandall, L.J., Daigh, C.A., Conard, K.R., Piekarczyk, M.S., *et al.* (2006). Derivation of human embryonic stem cells in defined conditions. Nature biotechnology *24*, 185-187.

Magini, P., Della Monica, M., Uzielli, M.L., Mongelli, P., Scarselli, G., Gambineri, E., Scarano, G., and Seri, M. (2012). Two novel patients with Bohring-Opitz syndrome caused by de novo ASXL1 mutations. American journal of medical genetics Part A *158A*, 917-921.

Rehimi, R., Nikolic, M., Cruz-Molina, S., Tebartz, C., Frommolt, P., Mahabir, E., Clement-Ziza, M., and Rada-Iglesias, A. (2016). Epigenomics-Based Identification of Major Cell Identity Regulators within Heterogeneous Cell Populations. Cell reports *17*, 3062-3076.

Schindelin, J., Arganda-Carreras, I., Frise, E., Kaynig, V., Longair, M., Pietzsch, T., Preibisch, S., Rueden, C., Saalfeld, S., Schmid, B., *et al.* (2012). Fiji: an open-source platform for biological-image analysis. Nat Methods *9*, 676-682.

Studier, F.W. (2005). Protein production by auto-induction in high density shaking cultures. Protein expression and purification *41*, 207-234.
